# Seroprevalence of ToRCH Pathogens in Southeast Asia

**DOI:** 10.3390/microorganisms9030574

**Published:** 2021-03-11

**Authors:** Franziska E. Fuchs, Maude Pauly, Antony P. Black, Judith M. Hübschen

**Affiliations:** 1 Department of Infection and Immunity, Luxembourg Institute of Health, L-4354 Esch-sur-Alzette, Luxembourg; FranziskaFuchs@outlook.de (F.E.F.); paulymaude@gmx.net (M.P.); 2 Lao-Lux-Laboratory, Institut Pasteur du Laos, P.O. Box 3560 Vientiane, Laos; a.black@pasteur.la

**Keywords:** Southeast Asia, ToRCH, pregnancy, infection

## Abstract

ToRCH is the acronym for several pathogens associated with pregnancy complications and sequelae in the unborn or newborn child. Particularly primary infections during pregnancy are associated with increased risk. Seroprevalence data of ToRCH infections are important, especially in countries with weak disease surveillance systems, to estimate immunity and vaccination levels, as well as exposure rates and thus the risk of infection during pregnancy. A systematic literature search spanning a 30-year time period was conducted to identify serosurveys on ToRCH pathogens in Southeast Asia. The 96 identified studies showed that some pathogens were well studied, while only limited data were available for others. Studies from the better-developed countries of the region were more abundant. Moreover, seroprevalence data were often limited to a certain geographical region within the country or to certain cohorts, there was an evident lack of recent serosurveys, and the study quality was often not adequate. Well-designed and area-wide serosurveys of ToRCH pathogens are clearly warranted. If combined with risk factor analysis, these studies may guide the development and implementation of effective measures for infection prevention, especially during pregnancy. In addition, educational programs for health care workers and for pregnant women during antenatal care are urgently needed.

## 1. Introduction

ToRCH is the acronym for several pathogens associated with pregnancy complications. ToRCH pathogens include *Toxoplasma gondii* (*T. gondii*), others (such as Varicella zoster virus—VZV and Primate erythroparvovirus 1—B19V), Rubella virus (RV), Cytomegalovirus (CMV), and Herpes simplex virus (HSV) [1,2] (Table 1). Primary infected pregnant women are mostly asymptomatic or show only mild symptoms. However, transplacental, perinatal or postnatal transmission of the pathogens can severely affect the unborn or newborn child. Sequelae include preterm birth, anomalies, stillbirth, fetal growth restriction, organ injuries, and chronic postnatal infections [3,4,5]. Usually, latent infections or virus reactivations of the mother do not pose a threat to the unborn child [6].

The global impact of congenital infections remains largely unknown. In developing countries, the burden is thought to be higher than in industrialized countries, because of coinfections of the mother or mother and child malnutrition [4]. In addition, inefficient or inexistent disease surveillance and knowledge gaps among healthcare workers (HCW) lead to underreporting and misdiagnosis [4,7]. Data on acute ToRCH infections in the included countries are rare. Disease surveillance systems for RV are established in all studied countries and for congenital rubella syndrome (CRS) in all but Malaysia [8,9], while surveillance for VZV infections seems to exist only in Malaysia and Thailand [10].

**Table 1:** Overview of important ToRCH pathogens. B19V = Primate erythroparvovirus 1; CMV = Cytomegalovirus; HSV = Herpes simplex virus; RV = Rubella virus; *T. gondii* = *Toxoplasma gondii*; VZV = Varicella zoster virus; CRS = congenital rubella syndrome.

Pathogen	Routes of Transmission Besides Transplacental Spread	Consequences for the Mother	Consequences for the Unborn or Newborn Child	Vaccination Available	Treatment Available	References
*T. gondii*	Ingestion of oocysts or tissue cysts via contaminated food, or from soil, cat feces etc.	Mostly asymptomatic; Possible complications: lymphadenopathy; myocarditis; pneumonia; hepatitis; encephalitis	Stillbirth; brain damages (intracranial calcifications; hydrocephalus; microcephaly; mental retardation); hepatic enlargement; ocular damages; subclinical infections with development of ocular lesions at later time point	No	Yes	[11,12]
VZV	Human-to-human transmission, respiratory or contact with lesions	Mostly harmless rash and mild flu-like symptoms; Possible complications: severe pneumonia and death	Intrauterine death; Congenital varicella syndrome (skin lesions; neurologic defects; eye diseases; skeletal anomalies); Neonatal varicella	Yes	Yes	[13,14]
B19V	Human-to-human transmission, respiratory, blood	Mostly asymptomatic; mild illness with fever; rash; malaise, headache; Possible complication: polyarthritis	Preterm delivery; miscarriage; severe anemia; nonimmune hydrops fetalis; meningoencephalitis	No	No	[5]
RV	Human-to-human transmission, respiratory	Mostly asymptomatic or mild illness with rash; Possible complication: polyarthritis	Spontaneous abortion; miscarriage; stillbirth; fetal growth restriction; CRS (small for infant age; hearing loss; cataract and heart defects)	Yes	No	[5,15]
CMV	Human-to-human transmission by body fluids, e.g., saliva, urine, blood	Mostly asymptomatic; Possible complications: fever; pharyngitis; lymphadenopathy; hepatosplenomegaly; arthralgia; rash	Mostly asymptomatic; Spontaneous abortion; fetal death or preterm birth; mental retardation; hearing loss; fetal growth restriction	No	Yes	[3,4,16]
HSV	Human-to-human transmission by contact with lesions	Mostly asymptomatic; Possible complications: Blistering; ulceration in the genital or oral region; fever; lymphadenopathy; systemic infection	Preterm delivery; spontaneous abortion; fetal death; cutaneous symptoms; brain damages (microcephaly; intracranial calcifications; encephalitis); Neonatal infection (skin, eye, central nervous system manifestations); systemic infection	No	Yes	[3,17,18]

In several Southeast Asian countries, the neonatal mortality rates (NMR) still rank among the highest worldwide. The upper-middle-income countries Malaysia and Thailand had NMR of 4.3 and 5.0, the lower-middle-income countries Cambodia and Laos reported NMR of 14.4 and 22.7 and only the high-income country Singapore reported a very low NMR of 1.1 per 1000 live births in 2018 [19]. It is likely that deaths resulting from congenital ToRCH infections as well as their complications (including pneumonia, pre-term birth, sepsis, and congenital abnormalities) contribute considerably to the high NMR [20].

Presence of immunoglobulin (Ig) G antibodies against a specific pathogen indicates past infection or vaccination. Consequently, IgG serosurveys represent an efficient approach to estimate immunity and vaccination levels, as well as exposure rates. When combined with socio-demographic and behavioral data, such studies allow the identification of risk factors (RFs) for infection and of vulnerable population groups, as well as the need for introducing new vaccines [21]. However, since most serosurveys are conducted in developed countries, the situation in developing countries is poorly understood [22].

To get an overview over the current situation in Southeast Asia, a systematic review of ToRCH serosurveys was conducted. Focus was put on Cambodia, Laos, Myanmar, Malaysia, Singapore, Thailand, and Vietnam, because of their geographic proximity and their social and economic disparities. Based on the available seroprevalence data, we assessed the risk of pregnant women to become infected and identified RFs for past infection.

## 2. Materials and Methods

### 2.1. Literature Search

**Figure 1:**
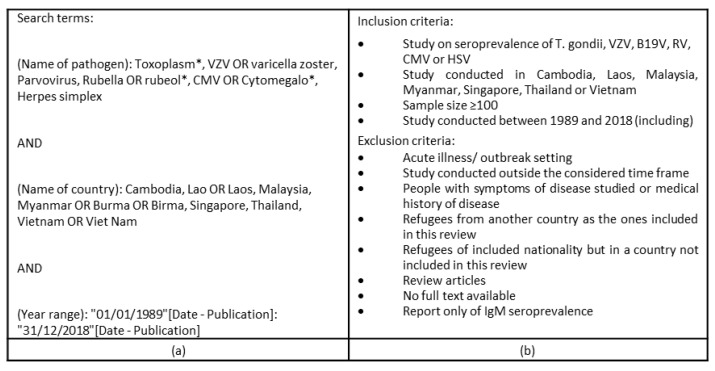
(**a**) PubMed search terms; (**b**) Inclusion and Exclusion criteria for the literature review.

A systematic literature review was conducted. The database PubMed was searched in January 2019 for studies published between 1 January 1989 and 31 December 2018. The search terms included Name of the pathogen (abbreviation as well as full name), Name of the country (different spellings if applicable) and the Year range (1 January 1989 to 31 December 2018) (Figure 1a). After removing duplicates, the titles of all identified studies were screened for their relevance. Only titles suggesting that ToRCH seroprevalence data were obtained were kept. If the abstracts of the selected articles met the inclusion criteria (Figure 1b), the full texts were screened if available. In a second step, the references quoted in these articles were checked and articles citing the retained studies were retrieved with Google Scholar to identify missed articles. The loose inclusion and exclusion criteria (Figure 1b) allowed drawing a representative picture of data availability and quality in the study area.

### 2.2. Data Processing

Overall and age-distributed seroprevalence, as well as 95% Confidence Intervals (95% CI) were extracted from the studies. If not available, a free online sample size calculator [23] was applied to estimate the 95% CI (https://www.surveysystem.com/sscalc.htm, 11 January 2019) using the given sample size and overall seroprevalence. If studies included more than one study cohort, the seroprevalence of each cohort was considered separately if it met the inclusion criteria (Figure 1b). If only age-distributed seroprevalence was available, the overall seroprevalence was calculated by dividing the number of seropositive participants by the total number of participants. Some studies reported combined seroprevalence for IgG and IgM. If possible, the IgG seroprevalence was calculated as mentioned above. If the differentiation between IgG and IgM data was not feasible, the overall IgM/IgG seroprevalence was shown as IgG seroprevalence but tagged accordingly. Since only asymptomatic cohorts without acute infections were considered (Figure 1b), the IgM/IgG seroprevalence largely reflects IgG seroprevalence. Finally, to obtain an estimate of the national seroprevalence of a pathogen, the seroprevalence range for the country was specified when there were more than three studies available in the country. If the same data on seroprevalence of a pathogen was reported in multiple studies, the data was considered only once.

### 2.3. Quality Criteria

The quality of the studies was evaluated by applying criteria of a recent review [24] after slight adaptation (Table S1). Quality criteria were, for example, information about study location and sample size, as well as sample size calculation and inclusion of ethical consideration and reporting bias. For each quality criteria fulfilled, a point was attributed to the study with a maximum of 17 achievable points. The quality criteria were not considered to be inclusion or exclusion criteria but were only used to assess the quality of research.

## 3. Results

**Figure 2:**
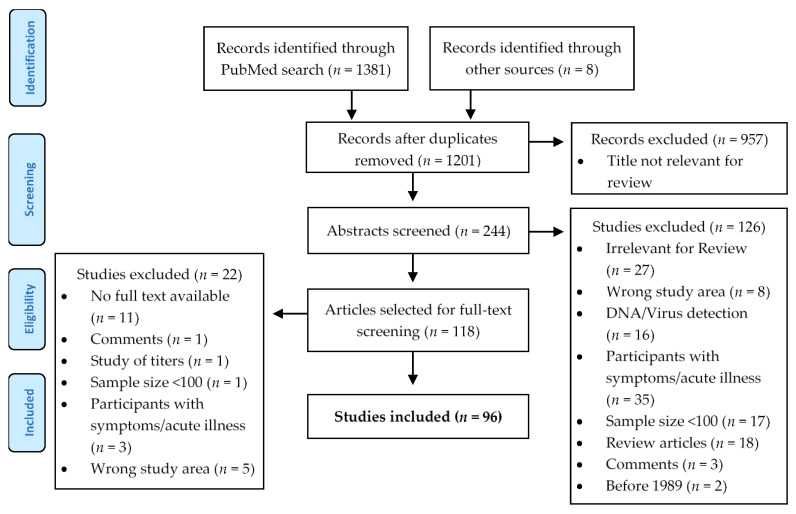
Flowchart of the identification process of eligible articles.

In total, 96 studies were eligible (see Figure 2 based on Moher et al. [25]):

37 on *T. gondii*, 7 on VZV, 5 on B19V, 15 on RV, 8 on CMV, 16 on HSV and 8 studies on multiple ToRCH pathogens. The studies contained 124 seroprevalence rates among different cohorts. The quality criteria scores ranged between 5 and 17 points with a mean of 10.6 points and with a higher score for more recent studies from 2010 to 2018 (9.2 vs. 12.7). The criteria “Overall result given” was fulfilled most often (*n* = 92), while only 14 studies provided the least fulfilled criteria “Sample size calculation done” (Table S1).

### 3.1. Toxoplasma Gondii

A high proportion of studies reported *T. gondii* seroprevalence (42/96). Five of these studies reported seroprevalence rates in more than one study population (Table 2).

**Table 2:** *Toxoplasma gondii* IgG serosurveys. * Only results on overall antibody seroprevalence (IgM + IgG) are available; Confidence Intervals (CI) in Italics are estimated as described in the Methods; CFT = Complement fixation test; ELISA = Enzyme-linked Immunosorbent Assay; IFAT = Immunofluorescent antibody test; LAT = Latex agglutination test; NA = not available; y = years.

	Study Location and Year	Study Population (*n*, Age Range)	IgG Seroprevalence in % (95% CI) *	Detection Method	*Comments* and/or Risk Factors for Seropositivity	Reference, Year Published
Cambodia	Phnom Penh, NA	Adults, (335, NA)	13.1 (*9.49–16.71*) *	Direct agglutination test (BioMérieux), Immunoenzymatic test (Platelia IgM and IgG, Sanofi Pasteur)		[26], 1999
	Nationwide, 2012	Women, (2150, 15–39y)	5.8 (4.7–7.0)	Multiplex Bead Assay		[27], 2016
Laos	Keoudom, NA	General population, (588, 3–70 y)	15.3 (*12.39–18.21*) *	CFT	Higher age	[28], 1992
Malaysia Range: 10.6–59.7	Kuala Lumpur, NA	Pregnant women, (219, 20–41 y)	39.7 (*33.25–46.21*)	ELISA (IgM- and IgG-NovaLisa, Dietzenbach, Germany)	Higher age; Low level of education and awareness; Parity (≥1); Lack of awareness of toxoplasmosis; Consumption of undercooked meat	[29], 2014
	Selangor, NA	Aborigines, (415, NA)	10.6 (*7.64–13.56*) *	IFAT		[30], 1994
	Kuala Lumpur, NA	Blood donors, (203, 18–65 y)	28.1 (*21.92–34.28*)	ELISA		[31], 2002
	Kuala Lumpur, 1994 to 2001	HIV-infected, (406, 17–74 y)	51.2 (*46.34–56.06*)	ELISA (AxSYM, Abbott Laboratories, USA)		[32], 2003
	Kuala Lumpur, 2001 to 2002	HIV-infected, (505, 17–71 y)	44.8 (42.64–51.76)	ELISA (AxSYM, Abbott Laboratories, USA)		[33], 2004
	Kuala Lumpur, 2002	HIV-infected, (301, 18–78 y)	41.2 (35.5–46.9)	ELISA (Trinity Biotech, Bray, Ireland)	Ethnicity (Malay)	[34], 2003
	Kuala Lumpur, 2002	Pregnant women, (200, 18–43 y)	39.0 (32.24–45.76)	ELISA (Trinity Biotech, Bray, Ireland)	Ethnicity (Malay)	[35], 2003
	Kuala Lumpur, 2000 to 2004	HIV-infected, (162, 1–85 y)	35.8 (*28.42–43.18*)	ELISA (Trinity Biotech, Bray, Ireland and Veda-lab, Alencon Cedex, France)		[36], 2005
	Kuala Lumpur, 2000 to 2004	Ocular patients, (161, 1–85 y)	31.1 (*23.95–38.25*)	ELISA (Trinity Biotech, Bray, Ireland and Veda-lab, Alencon Cedex, France)		[36], 2005
	NA, NA	Worker, (198, NA)	44.9 (*37.97–51.83*)	IFAT		[37], 2008
	Kuala Lumpur, 2007 to 2008	Renal patients, (247, 21–89 y)	46.6 (40–52)	ELISA (IgM and IgG, Trinity Biotech, New York, USA)	Ethnicity (Malay); Marital status (married); Low level of education	[38], 2011
	Kuala Lumpur, 2009	Oncology patients, (129, 15–88 y)	38.8 (*30.34–47.16*)	ELISA	Living in rural areas; Consumption of undercooked meat and/or history of blood transfusion	[39], 2010
	Peninsular, 2007 to 2010	Indigenous, (495, 1–82 y)	31.0 (26.9–35.1)	ELISA (IgM and IgG, Trinity Biotech, New York, NY, USA)	Age (>12 y); domestic use of untreated river and mountain water; Close contact with pets	[40], 2011
	Kuala Lumpur, 2010	Ocular patients, (493, 2–90 y)	25.0 (21.0–29.0)	ELISA (Trinity Biotech, New York, USA)	Higher age; Ethnicity (Malay)	[41], 2012
	Kuala Lumpur, 2011	Patients with Schizophrenia, (144, NA)	37.5 (*29.59–45.41*)	ELISA (Platelia Toxo IgG ELISA BioRad, USA)	Age (>40 y); Ethnicity (Malay)	[42], 2012
	Kuala Lumpur, 2011	Healthy patients, (144, NA)	34.0 (*26.26–41.74*)	ELISA (Platelia Toxo IgG ELISA BioRad, USA)		[42], 2012
	Selangor, NA	Patients with Schizophrenia, (101, 18–65 y)	51.5 (*41.75–51.5*)	ELISA (IBL Company, Hamburg, Germany)		[43], 2015
	Pangkor Island, NA	General population, (298, 1–80 y)	59.7 (54.13–65.27) *	ELISA (IgM and IgG, Trinity Biotech, USA)	Gender (female); Ethnicity (Malay)	[44], 2014
	Kuala Lumpur, 2012 to 2013	Pregnant women, (281, NA)	33.5 (*27.98–39.02*)	ELISA (Platelia Toxo IgM and IgG, BioRad, USA)		[45], 2014
	NA, 2012 to 2013	Prison inmates, (303, NA)	39.3 (*33.8–44.8*)	ELISA (Platelia Toxo IgM and IgG, BioRad, USA)	Age (>40 y); HIV status (positive); Drug abuse history	[46], 2016
	Selangor, Klang Valley, 2013 to 2014	Veterinary personnel, pet owner, (312, 17–64 y)	18.3 (*14.01–22.59*)	ELISA (IgG-NovaLisa, Dietzenbach, Germany)	Age (≥30 y); Working duration (>10 y)	[47], 2015
Myanmar	Yangon, NA	Pregnant women, (215, 18–45 y)	30.2 (*24.09–36.37*)	ELISA (IgM- and IgG-NovaLisa, Dietzenbach, Germany)		[29], 2014
	Thailand-Myanmar-Border, 2014 to 2015	Pregnant women, (199, 16–46 y)	31.7 (25.6–38.4)	ELISA (IgM and IgG, Novatec, Dietzenbach Germany)	Parity (≥3)	[48], 2017
Singapore	Singapore, 1997 to 1998	Pregnant women, (120, NA)	17.2 (*10.45–23.95*)	IFAT		[49], 2000
	Singapore, 2006 to 2011	HIV-infected, (771, NA)	23.7 (*20.7–26.7*)	NA		[50], 2013
Thailand Range: 2.6–53.7	Bangkok, NA	Pregnant women, (468, NA)	12.6 (*9.59–15.61*)	LAT (Toxotest MT Eiken, Japan)		[51], 1991
	Bangkok, 1992 to 1995	Pregnant women, (300, 14–40 y)	13.7 (*9.81–17.59*)	ELISA (TOXOELISA II, Biowhittaker, USA)		[52], 1997
	Samut Sakhon, 1996	Pregnant women, (1200, NA)	13.2 (*11.28–15.12*)	Sabin-Feldman Dye Test	Consumption of undercooked meat	[53], 1998
	Samut Sakhon, NA	Pregnant women, (300, 14–41 y)	21.7 (*17.04–26.36*)	Sabin-Feldman Dye Test		[54], 1999
	Bangkok, 1997 to 1998	General population, (163, 2–89 y)	3.1 (0.44–5.76) *	LAT (Toxo Check, Eiken Chemical Co., Ltd., Japan)		[55], 2000
	Loei Province, 1997	Blood donors, (345, 17–56 y)	4.1 (*2.01–6.19*)	ELISA	Gender (male)	[56], 2000
	Bangkok, 1997 to 1999	HIV-infected pregnant women, (838, NA)	53.7 (*50.32–57.08*)	ELISA (Platelia Toxo IgG, Sanofi Diagnostics Pasteur, France)	HIV status (positive)	[57], 2001
	Bangkok, 1997 to 1999	Pregnant women, (831, NA)	5.3 (*3.78–6.82*)	ELISA (Platelia Toxo IgG, Sanofi Diagnostics Pasteur, France)		[57], 2001
	Bangkok, 1999 to 2000	Pregnant women, (200, NA)	13.6 (*8.85–18.35*)	ELISA		[58], 2001
	Bangkok, NA	General population, temple residents (327, 2–75 y)	6.4 (*3.75–9.05*)	Sabin-Feldman Dye Test	Cat ownership	[59], 2003
	Khon Kaen, 2009 to 2012	Women, (493, 21–81 y)	2.6 (1.2–4.0) *	LAT (TOXOTEST-MT Eiken, Eiken-Kagaku, Tochigi, Japan)		[60], 2013
	Songhkla Province, Hat Yai, 2009 to 2010	Pregnant women, (640, 15–45 y)	21.6 (18.5–24.9)	ELISA (IgG-Trinity Biotech, New York)	Age (≥36 y); Living outside Songkhla province; Contact with cats; Drinking unclean water	[61], 2011
	Songhkla Province, 2009 to 2010	HIV-infected, (300, 21–78 y)	36.3 (*30.86–41.74*)	ELISA (IgG-NovaLisa, Dietzenbach, Germany)	Gender (male)	[62,63], 2013, 2015
	Songhkla Province, Hat Yai, 2012 to 2013	Pregnant women, (760, 14–47 y)	22.0 (19.0–25.0)	ELISA (IgG- and IgM- Trinity Biotech, New York)	Age (≥26 y); Working as a laborer; Drinking unclean water	[64], 2014
Vietnam Range: 4.2–11.2	Ho Chi Minh City, 1996	HIV-positive injecting drug users, (235, 24–57 y)	9.0 (*5.34–12.66*)	ELISA (IgM and IgG; Behring)		[65], 1999
	Nghe An, Lao Cai and Tien Giang provinces, 2006	General population, (650, NA)	4.2 (1.78–4.62)	Sabin-Feldman Dye Test		[66], 2008
	Ho Chi Minh City, NA	Drug addicted, (300, 18–53 y)	7.7 (*4.68–10.72*)	ELISA (Platelia Toxo IgG, BioRad)		[67], 2003
	Ho Chi Minh City, NA	HIV-negative adults, (150, NA)	6.5 (*2.55–10.45*)	ELISA (Platelia Toxo IgG, BioRad)		[67], 2003
	NhaTrang, NA	Pregnant women, (300, 18–43 y)	11.2 (*7.63–14.77*)	ELISA (Platelia Toxo IgG, BioRad)		[67], 2003

#### 3.1.1. Seroprevalence by Country

Two studies from Cambodia investigated women of childbearing age (seroprevalence 5.8% [27]) and adults (13.1% [26]).

For Laos, the only available study dated back to the early 1990s and the seroprevalence was relatively low (15.3%) in the general healthy population [28].

Nearly half of the studies (19/42) were from Malaysia and the seroprevalence ranged from 10.6% [30] to 59.7% [44]. Most (*n* = 17) of the 21 seroprevalence rates mentioned in the 19 studies exceeded 30.0%. The seroprevalence among pregnant women ranged between 33.5% [45] and 39.7% [29] with an average of 37.4% and a median of 39.0%. With few exceptions, human immunodeficiency virus (HIV)-infected individuals had higher seroprevalence rates, reaching up to 51.2% (average 43.3%, median 43.0%) [32]. Most studies (15/19) were conducted in Kuala Lumpur or its periurban area.

The two regionally limited studies from Myanmar targeted pregnant women and the seroprevalence ranged from 30.2% [29] to 31.7% [48]. 

The two studies from Singapore showed a higher rate in the HIV-infected cohort (23.7%) [50] than in pregnant women (17.2%) [49]. 

Many studies were from Thailand (14/42) covering diverse study cohorts with a wide range of reported seroprevalence rates (2.6% [60] to 53.7% [57]). Most rates (9/14) were below 15%. The highest were found for HIV-infected individuals (53.7% [57] and 36.3% [62,63]). In contrast, a low seroprevalence rate (<5% [55,56,60]) was reported for healthy individuals. Seroprevalence rates for pregnant women ranged from 5.3% [57] to 22.0% [64]. Again, most (9/14) of the studies were done in the capital or the surrounding provinces. In the North of Thailand, studies reported seroprevalence values as low as 2.6% and 4.1% in healthy adults [56,60] compared to higher rates of 21.6% and 22.0% in the South in healthy pregnant women [61,64].

Low seroprevalence rates of *T. gondii* antibodies were reported in the three studies from Vietnam, irrespective of study populations (range: 4.2% [66] to 11.2% [67]). The highest seroprevalence in Vietnam was reported in pregnant women [67]. Most studies were from the South of Vietnam, only one study included a population from the North [66].

For 15 of the reported seroprevalences, no study year was available. The remaining studies were conducted between 1992 [52] and 2015 [48] with no notable change in seroprevalence over time. The studies on *T. gondii* seroprevalence included different populations with the majority focusing on healthy pregnant women (15/42) or HIV-infected individuals (8/42), with the seroprevalence mostly being higher among HIV-infected people. While the large majority (28/42) of the studies used a commercial ELISA kit, other detection methods (e.g., Sabin-Feldman Dye Test or Immunofluorescent antibody test) were used especially in studies published before 2000. Similar seroprevalence rates were found using the different test methods in the respective countries and study populations.

#### 3.1.2. Risk Factors for Seropositivity

Only 21 studies included an assessment of RFs for seropositivity to *T. gondii* antibodies.

Nine studies conducted in Laos, Malaysia or Thailand reported that higher age (e.g., older than 30 years [47,61] or 40 years [42,46]) was statistically significant associated with *T. gondii* seropositivity. This RF did not seem to be restricted to a specific study population. Identified RFs were often indirectly linked to age such as parity [29,48] and marital status [38]. 

Statistically significant RFs identified in Thailand and Malaysia were HIV positivity [46,57], consumption of undercooked or raw meat [29,39,53], use of untreated water [40,61,64], contact to cats [40,59,61] and low level of knowledge or awareness [29,38]. In Malaysia, Malay ethnicity was a frequently reported RF for *T. gondii* seropositivity [34,35,38,41,42,44].

### 3.2. Varicella Zoster Virus

Of the 96 identified studies, 11 investigated VZV IgG seroprevalence (Table 3).

**Table 3:** VZV IgG serosurveys. Confidence Intervals (CI) in Italics estimated as described in the Methods, HCW = Healthcare workers; EIA = Enzyme immunoassay; y = years; ELISA = Enzyme-linked Immunosorbent Assay; NA = not available.

	Study Location and Year	Study Population (*n*, Age Range)	IgG Seroprevalence in % (95% CI)	Detection Method	*Comments* and/or Risk Factors for Seropositivity	Reference, Year Published
Cambodia	NA					
Laos	Vientiane Capital, Huaphan Province, Boulhikhamxay Province, 2013	HCW, (1128, 15–69 y)	95.0 (*93.73–96.27*)	ELISA (Euroimmun)	Early life exposure (*15–24y age group: already 94.1% positive*)	[68], 2015
Malaysia	Kuala Lumpur, NA	HIV-infected, (232, 32–43 y)	86.6 (*82.22–90.98*)	ELISA (Siemens Enzygnost, Siemens Healthcare GmbH, Germany)		[69], 2017
Myanmar	NA					
Singapore Range: 55.3–91.7	Singapore, 2000 to 2005	Military men, (2189, 16–36 y)	76.0 (*74.21–77.79*)	ELISA		[70], 2007
	Singapore, 2008 to 2010	General population, (1200, 1–17 y)	55.3 (52.5–58.1)	EIA (Euroimmun AG, Germany)	Higher age; Ethnicity (Chinese)	[71], 2014
	Singapore,2009 to 2014	HCW, (6701, NA)	91.7 (*91.04–92.36*)	ELISA (Euroimmun Medizinische Labordiagnostika AG, Germany)	Higher age; Ethnicity (Chinese); HCW in nursing vocation	[72], 2015
Thailand Range: 52.8–92.0	Bangkok, 1994	General population, (559, 4M-77 y)	61.4 (*57.36–65.44*)	ELISA (Enzygnost, Behringwerke, Germany)	Higher age	[73], 1997
	Bangkok, Chiang Mai, Khoen Kaen, Had Yai, 1997 to 1998	General population, (2093, 9M-29 y)	52.8 (50.6–54.9)	ELISA (Enzygnost, Dade Behring, Marburg Germany)	Region (North); Central/South: Seroprevalence notably lower in rural areas; Higher age	[74], 2001
	Bangkok, 1998 to 2000	Healthy children, blood donors, (350, NA)	64.6 (*59.59–69.61*)	ELISA (Human, Germany)	Higher age; Increasing number of family members	[75], 2005
	Bangkok, 2006 to 2007	Medical students, (237, 20–38 y)	82.3 (*77.44–87.16*)	EIA		[76], 2009
	Bangkok, 2008 to 2009	Medical students, (374, 18–25.8 y)	92.0 (*89.25–94.75*)	ELISA (Wiesbaden, Germany)		[77], 2012
Vietnam	Ho Chi Minh City, 1996	Intravenous drug users, (235, 24–57 y)	99.0 (*97.73–100.27*)	ELISA (Behring, Germany)		[65], 1999

#### 3.2.1. Seroprevalence by Country

There were no studies on VZV seroprevalence from Cambodia and Myanmar and the only article from Laos reported a seroprevalence of 95.0% in HCW from northern and central provinces with high rates (94.1%) already in the 15- to 24-year-olds [68]. 

The only study from Malaysia reported a seroprevalence of 86.6% among HIV-infected patients in Kuala Lumpur [69]. Likewise, for the only study from Vietnam, 99.0% of HIV-infected drug users from Ho Chi Minh City had anti-VZV antibodies [65].

In the three studies from Singapore, the seroprevalence ranged from 55.3% [71] to 91.7% [72]. The high rate among HCW (91.7%) [72] was comparable to the situation among Lao HCW [68]. In contrast, a considerably lower seroprevalence (55.3%) was found among children and adolescents [71]. 

Nearly half of the studies were from Thailand (5/11) and all except one were conducted in Bangkok. The seroprevalence ranged from 52.8% [74] to 92% [77]. High seroprevalence rates were reported for medical students (92% [77] and 82.3% [76]) and the low rates for healthy children and adults (52.8% [74] to 64.6% [75]).

All studies in the region reported VZV IgG seroprevalence rates exceeding 50% (Table 3). The earliest study was conducted in 1994 [73] and the most recent was published in 2015 [72]. Irrespective of the limited amount of data, seroprevalence rates did not vary over the years in comparable study populations. Most studies (8/11) included HCW or the general population with HCW showing the highest VZV IgG seroprevalence.

Regardless of the study year, all identified studies used enzyme immunoassays (EIA) for detection of VZV IgG antibodies.

#### 3.2.2. Risk Factors for Seropositivity

Several studies suggested that VZV IgG seroprevalence increases with age. In fact, the lowest seroprevalence was found among children aged less than 4 years and 1- to 6-year-olds (11.3% [74] and 34.5% [71], respectively). Moreover, five of the studies from Singapore and Thailand identified increasing age as a statistically significant RF for VZV IgG seropositivity [71,72,73,74,75].

The highest seroprevalence rates were found in HCW [68,72] and in HIV-infected individuals [65,69]. As for *T. gondii*, some studies suggested ethnicity as RF. Singaporean studies identified highest seroprevalence values in participants with Chinese ethnicity and lowest in participants with Indian ethnicity [71,72]. 

One study suggested a higher seroprevalence in the more temperate regions of the North of Thailand than in the more humid regions in the South and an influence of population density with notably lower VZV IgG seroprevalence in rural compared to urban settings in the South [74].

### 3.3. Primate Erythroparvovirus 1

Six out of 96 studies investigated anti-B19V IgG seroprevalence (Table 4).

**Table 4:** Primate erythroparvovirus 1 IgG serosurveys. Confidence Intervals (CI) in Italics estimated as described in Methods; ELISA = Enzyme-linked Immunosorbent Assay; NA = not available; y = years.

	Study Location and Year	Study Population (*n*, Age Range)	IgG Seroprevalence in % (95% CI)	Detection Method	*Comments* and/or Risk Factors for Seropositivity	Reference, Year Published
Cambodia	NA					
Laos	NA					
Malaysia	Kuala Lumpur, 1999 to 2000	Blood donors, undergraduate students, patients, (800, 6M–81 y)	37.6 (*34.24–40.96*)	ELISA (Biotrin, Dublin, Ireland)	Higher age	[78], 2002
Myanmar	NA					
Singapore	Singapore, 1993	General population, (600, 6M–50 y)	16.2 (*13.25–19.15*)	ELISA	Higher age	[79], 1994
	Singapore, 1997 to 1998	Pregnant women, (120, NA)	30.0 (*21.8–38.2*)	ELISA	Higher age	[49], 2000
Thailand Range: 10.9–20.2	Bangkok, Songkhla Province, 1998 to 1999	Children and blood donors, (129, 0–51 y)	20.2 (*13.24–27.08*)	ELISA (Genzyme Virotech GmbH, Germany)	Higher age	[80], 2000
	Bangkok, 1998 to 1999	Immunocompromised children, (106, 1–15 y)	16.0 (*9.02–22.98*)	ELISA (Genzyme Virotech GmbH, Russelsheim, Germany)		[81], 2000
	Bangkok, 1999 to 2000	Undergraduate students, (128, 18–24 y)	10.9 (*5.53–16.35*)	ELISA (Genzyme Virotech GmbH, Russelsheim, Germany)		[82], 2003
Vietnam	NA					

#### 3.3.1. Seroprevalence by Country

No studies from Cambodia, Laos, Myanmar, and Vietnam and only one from Malaysia were identified.

The latter study included blood donors, undergraduate students and various immunocompetent patients recruited between 1999 and 2000 in Kuala Lumpur and found an overall seroprevalence of 37.6% [78]. 

Half of the studies (3/6) were from Thailand, where B19V IgG seroprevalence rates ranged between 10.9% [82] and 20.2% [80]. Two studies focused on younger cohorts, while one study included children as well as blood donors up to the age of 51 years [80]. 

In the two studies from Singapore, seroprevalence rates of 16.2% and 30.0% were reported in a healthy study population [79] and in pregnant women [49], respectively. 

Recent studies were missing. Before 2000, B19V IgG seroprevalence did not exceed 40% [78]. Due to the small amount of data, no regional trends could be identified. Studied populations included mainly healthy persons such as students, blood donors or a general healthy population [78,79,80,82]. Three studies focused on various healthy individuals [79,80,82] and two on vulnerable study populations (i.e., pregnant women and immunocompromised children [49,81]). Regardless of the study year or the country, all studies used EIAs for the detection of anti-B19V IgG.

#### 3.3.2. Risk Factors for Seropositivity

Due to differing study designs and the limited number of studies, it was difficult to extract trends or identify RFs. 

In Singapore, highest rates were found in pregnant women and lowest rates in healthy individuals. Whether there is a statistically significant difference in seroprevalence between different age groups remains unknown. In one study, seropositivity increased with age, but remained below 70% even in older participants [78].

### 3.4. Rubella Virus

Eighteen studies covered anti-RV IgG seroprevalence (Table 5). Four of these studies investigated seroprevalence rates for multiple study populations.

**Table 5:** Rubella IgG serosurveys. Confidence Intervals (CI) in Italics estimated as described in Methods, ANC = Antenatal care; EIA = Enzyme immunoassay; ELISA = Enzyme-linked Immunosorbent Assay; HCW = Healthcare workers; MEIA = Microparticle EIA; NA = not available; RCV = Rubella containing vaccine; y = years.

	Study Location and Year	Study Population (*n*, Age Range)	IgG Seroprevalence in % (95% CI)	Detection Method	Comments and/or Risk Factors for Seropositivity	Reference, Year Published
Cambodia	Nationwide, 2012	Women, (2154, 15–39 y)	73.3 (70.5–76.1)	ELISA (Enzygnost, Siemens, Germany)	*Study prior to RCV introduction;* Age (15–19 y); Living area (rural)	[83], 2015
Laos Range: 43.6–86.2	Vientiane Capital, 2007 to 2008	School children, (411, 6–12 y)	43.6 (*38.8–48.4*)	EIA (Denka Seiken, Japan)	*Study prior to RCV introduction;* Gender (girls); Age (6 y); Place of birth (hospital)	[84], 2011
	Vientiane Capital, Huaphan, Boulhikhamxay, 2013	HCW, (1128, 15–69 y)	86.2 (*84.2–88.2*)	ELISA (Euroimmun)	Childless	[68], 2015
	Nationwide, 2014	General population, (2135, 1–2y, 5–81 y)	75.4 (75.3–75.5)	ELISA (Enzygnost, Siemens Healthcare Diagnostics)	Not with been included in the SIA 2011	[85], 2018
Malaysia	Kuala Lumpur, 2001 to 2002	Pregnant women, (414, 15–45 y)	92.3 (*89.7–94.9*)	EIA (EIAgen, Italy)		[86], 2005
	Selangor, 2005	Pregnant women, (500- 16–42 y)	88.6 (*86.8–92.3*)	MEIA (AxSYM)	Laborer; No history of vaccination	[87,88], 2008, 2013
Myanmar	NA					
Singapore Range: 71.7–88.5	Singapore, 1993	General population, (909, NA)	71.7 (*68.77–74.63*)	MEIA (Abbott)		[89,90], 2010
	Singapore, 1998	General population, (928, NA)	80.2 (*77.64–82.76*)	MEIA (Abbott)		[89,90], 2010
	Singapore, 2004	General population, (4153, 18–74 y)	84.0 (82.9–85.1)	MEIA (Abbott)		[89,91], 2010, 2015
	Singapore, 2010	General population, (3293, 18–79 y)	85.0 (83.7–86.2)	Chemiluminescent microparticle immunoassay (Abbott Park, Ireland)	Ethnicity (permanent residents); higher age (among women)	[91], 2015
	Singapore, 2008 to 2010	Children, (1200, 1–17 y)	88.5 (86.6–90.2)	Chemiluminescent immunoassay (Abbott Architect, Abbott Laboratories, USA)	Ethnicity (Malay)	[91,92], 2015, 2013
Thailand Range: 74.7–89.4	Bangkok, 1992 to 1995	Pregnant women, (300, 14–40 y)	85.7 (*81.74–89.66*)	ELISA (Rubelisa II, Biowhittaker, USA)		[52], 1997
	Khon Kaen, 2004	Pregnant women, (150, 15–40 y)	74.7 (*67.6–81.6*)	ELISA		[93], 2005
	Chiang Rai, Udon Thani, Chon Buri, Nakhon Si Thammarat, 2004	General population, (899, 0–59 y)	89.0 (86.6–91.0)	ELISA (RE57081; IBL)		[94], 2009
	Pathum Thani 2006 to 2007	Medical students, (237, 20–38 y)	88.2 (*84.1–92.3*)	EIA		[76], 2009
	Chiang Mai, 2011	HIV-infected, (500, 36–48 y)	84.6 (*81.4–87.8*)	ELISA (Enzygnost, Siemens, Marburg, Germany)		[95], 2016
	Chiang Mai, 2011	Adults, (132, 30.5–59 y)	89.4 (*84.2–94.7*)	ELISA (Enzygnost, Siemens, Marburg, Germany)		[95], 2016
	Bangkok, 2014	Women, (289, 28–40 y)	87.2 (*83.4–91.0*)	ELISA (Euroimmun, Lübeck, Germany)		[96], 2018
Vietnam	Nha Trang, 2009 to 2010	Pregnant women, (1988, 17–45 y)	71.1 (*69.1–73.1*)	EIA (Mini VIDAS)	*Study prior to RCV introduction*; *Study used cord blood*; Young age; Primipara; Increased no. of ANC visits; Preterm delivery	[97], 2014

#### 3.4.1. Seroprevalence by Country

All studied countries currently use rubella-containing vaccine (RCV) for infants [98]. 

The only study from Cambodia was conducted before the introduction of RCV in the country [83,99] and revealed an average seroprevalence of 73.3% among women aged 15 to 39 years [83]. 

In the three studies from Laos, anti-RV IgG seroprevalence ranged from 43.6% [84] to 86.2% [68] among children and HCW. The earliest study was conducted before the introduction of the RCV [84]. Seroprevalence in children showed an increase from 43.6% in 6- to 12-year old [84] to 90.2% in an age group of 5- to 14-year old children [85].

The three studies from Malaysia were conducted after introduction of RCV [86,87,88,99] and reported seroprevalence rates in pregnant women from Kuala Lumpur ranged between 88.6% [87,88] and 92.3% [86]. 

In the four studies from Singapore, seroprevalence rates ranged from 71.7% [89,90] to 88.5% [91,92] in healthy adults or children. The studies were conducted after introduction of RCV [89,90,91,92,99] and seropositivity seemed to increase over the years. 

The six studies from Thailand were also conducted after introduction of RCV [52,76,93,94,95,96] and showed a seroprevalence range from 74.7% [93] to 89.4% [95] in varying study populations of pregnant women, healthy adults, HIV-infected individuals, and medical students. 

The only study from Vietnam was done before introduction of RCV [97,99] and found an overall seroprevalence of 71.1% in pregnant women [97]. 

While RV IgG seroprevalence was generally high in all locations, the situation in certain countries (i.e., Laos, Myanmar, Cambodia, and Vietnam) is less clear due to the limited number of studies. All studies, except one including only unvaccinated children [84], reported seroprevalence rates above 70% in the general population [85,89,90,91,94] and in pregnant women or women of childbearing age [52,83,86,87,88,93,96,97]. 

The studies used various types of immunoassays, but seroprevalence results did not seem to vary greatly between these assays.

#### 3.4.2. Risk Factors for Seropositivity

In contrast to the other pathogens, most studies reported RFs for susceptibility to RV rather than for seropositivity. 

As expected, several studies conducted before introduction of RCV found that younger age groups [83,84,97] were more likely to be susceptible to RV infection, while studies conducted after RCV introduction found that seroprevalence rates decreased with age [68,85,86,91]. 

Singaporean studies identified Malay ethnicity or permanent residency in Singapore as RF for susceptibility to RV [91,92]. One study identified occupation as a “worker” as a statistically significant RF for susceptibility [87], while others reported missing history of vaccination [87,88] or not being included in the Supplementary Immunization Activity (SIA) of 2011 in Laos [85] as RFs.

### 3.5. Cytomegalovirus

Overall, 14 studies on CMV seroprevalence were identified (Table 6).

**Table 6:** CMV IgG serosurveys. * Only results on overall antibody seroprevalence (IgM + IgG) are available; Confidence Intervals (CI) in Italics estimated as described in Methods; EIA = Enzyme immunoassay; ELISA = Enzyme-linked Immunosorbent Assay; MEIA = Microparticle EIA; NA = not available; y = years.

	Study Location and Year	Study Population (*n*, Age Range)	IgG Seroprevalence in % (95% CI) *	Detection Method	*Comments* and/or Risk Factors for Seropositivity	Reference, Year Published
Cambodia	NA					
Laos	NA					
Malaysia Range: 84.0–97.6	Kuala Lumpur, NA	Blood donors, (172, 18–47 y)	97.6 (*95.31–99.89*)	MEIA (Abbott Axsym System)		[100], 2006
	Kuala Lumpur, NA	HIV-infected, (232, 32–43 y)	96.1 (*93.61–98.59*)	Immunoassay (Elecsys, (Roche, Switzerland)		[69], 2017
	Nationwide, 2007 to 2008	Pregnant women, (125, NA)	84.0 (*77.57–90.43*)	ELISA (DRG Instruments GmbH, Marburg, Germany)		[101], 2011
Myanmar	NA					
Singapore	Singapore, 1997 to 1998	Pregnant women, (120, NA)	87.0 (*80.98–93.02*)	ELISA	Higher age (*no statistically significant trend*); Ethnicity (Not-Singaporeans)	[49], 2000
	Singapore, 2006 to 2011	HIV-infected, (753, NA)	96.8 (*95.54–98.06*)	NA		[50], 2013
Thailand Range: 52.4–100	Khon Kaen, 1990	Blood donors, (359, 17–59 y)	93.3 (90.78–95.89) *	ELISA (Abbott Laboratories)	Higher age (*no statistically significant trend*)	[102], 1993
	Bangkok, 1999 to 2000	Pregnant women, (200, NA)	79.7 (*74.13–85.27*)	ELISA		[58], 2001
	Bangkok, 1992 to 1995	Pregnant women, (300, 14–40 y)	100.00	ELISA (Biowhittaker, USA)		[52], 1997
	Bangkok, 1997	Blood donors, (380, 17–50 y)	71.8 (*67.28–76.32*)	ELISA		[103], 1999
	Bangkok, 1997	Pregnant women, (209, 15–45 y)	90.9 (*87–94.8*)	ELISA	Higher age	[103], 1999
	Bangkok, 1998	Blood donors, (441, 18–55 y)	52.4 (*47.72–57.04*)	ELISA		[104], 2001
	NA, NA	Mothers, (2101, NA)	86.53 (*85.07–87.99*)	Immunoassay (Abbott Diagnostics, Abbott Park, IL, USA)	*Study used cord blood*	[105], 2013
	Bangkok, 1997	Blood donors, (303, 16–56 y)	97.0 (95.08–98.92) *	ELISA (Enzygnost, Behring, Germany)	Sex (female)	[106], 1998
	Bangkok, 1995, 1997	Students, (172, 17–25 y)	86.0 (*80.81–91.19*)	ELISA (Enzygnost, Behring, Germany)	Sex (female)	[106], 1998
	Bangkok, 1997	Pregnant women, (100, 15–40 y)	100.0	ELISA (Enzygnost, Behring, Germany)		[106], 1998
	Bangkok, 1990	Blood donors, (2196, NA)	97.3 (96.62–97.98) *	EIA (Abbott)		[107], 1992
Vietnam	Ho Chi Minh City, 1996	Intravenous drug users (235, 24–57 y)	100.0	ELISA (Behring)		[65], 1999

#### 3.5.1. Seroprevalence by Country

No studies were found for Cambodia, Laos, or Myanmar. The seroprevalence reported in the three studies from Malaysia ranged between 84.0% [101] to 97.6% [100] with the lowest rate found among pregnant women [101]. In contrast to the other pathogens, the study locations covered not only Kuala Lumpur, but also the surrounding area as well as other regions. 

In the two studies from Singapore, the seroprevalence values were 87.0% [49] and 96.8% [50], with the lowest seroprevalence also reported for pregnant women [49]. 

Eight studies were identified for Thailand, where the seroprevalence ranged between 52.4% [104] and 100% [52,106]. Most study populations included pregnant women or blood donors (7/9) and only one study investigated students [106]. The lowest seroprevalence in Thailand of 52.4% was reported in blood donors [104]. Highest seroprevalence was reported among pregnant women [52,106]. The seroprevalence in blood donors varied widely from 52.4% [104] to 97.3% [107] and among pregnant women from 79.7% [58] to 100% [52,106]. 

One study from Vietnam conducted in 1996 included HIV-infected drug users in Ho Chi Minh City and showed an overall seroprevalence of 100% [65]. 

Seroprevalence was high in all countries and except for one study investigating Thai blood donors [104], rates were always above 70%. Most studies provided data for the years between 1990 and 2000 and only two studies were conducted later [50,101]. Most studies involved pregnant women (6/14) or blood donors, as representatives of a healthy population (6/14). For one study, no detection method was mentioned [50].

#### 3.5.2. Risk Factors for Seropositivity

Few RFs for seropositivity to CMV were identified. Although some studies noted an increase of seropositivity with age, this difference was not statistically significant [49,102]. One study in Thailand identified female sex as RF for seropositivity and related it to a stronger role of women in childcare [106]. 

Similar to studies of other pathogens from Singapore, ethnicity was identified as RF for seropositivity (Non-Singaporean ethnicity) [49].

### 3.6. Herpes Simplex Virus

In total, 19 studies were identified for HSV. The studies included seven seroprevalence rates for HSV-1, 16 for HSV-2 and one for unclassified HSV (Table 7).

**Table 7:** HSV IgG serosurveys. Confidence Intervals (CI) in Italics estimated as described in Methods; ELISA = Enzyme-linked Immunosorbent Assay; FSW = Female sex worker; HSV = Herpes simplex virus; MSM = Men who have sex with men; STI = sexually transmitted infection; NA = not available; y = years.

	Study Location and Year	Study Population (*n*, Age Range)	IgG Seroprevalence in % (95% CI)	Detection Method	*Comments* and/or Risk Fac tors for Serop Ositivity	Reference, Year Publ ished
(A) HSV-1						
Cambodia	NA					
Laos	NA					
Malaysia	Kuala Lumpur, NA	HIV-infected, (232, 32–43)	70.7 (*64.84–75.56*)	ELISA (HerpeSelect, Focus Diagnostics, Cypress, CA, USA)		[69], 2017
Myanmar	NA					
Singapore	Singapore, 2003 to 2004	Sex workers, (300, 22–70 y)	76.7 (*71.92–81.48*)	ELISA (HerpeSelect 1, Focus Diagnostics, Cypress, CA 90630, USA)		[108], 2006
	Singapore, 2003 to 2004	Attendees in STI clinic, (400, 15–80 y)	55.8 (*50.93–60.67*)	ELISA (HerpeSelect 1, Focus Diagnostics, Cypress, CA 90630, USA)	Higher age	[109], 2006
Thailand Range: 56.5–91.0	Phitsanulok, 1991	Male army conscripts, (1115, NA)	77.0 (74.4–79.4)	Immunoblot		[110,111], 1998, 1999
	Chiang Rai Province, 1991 to 1994	FSW, (500, NA)	91.0 (*88.49–93.51*)	Immunoblot		[112], 1999
	Bangkok, 2006 to 2010	MSM, (1744, 18–56 y)	56.5 (*54.17–58.83*)	ELISA (HerpeSelect 1, Focus Diagnostics, Cypress, CA 90630, USA)		[113], 2013
Vietnam	Ho Chi Minh City, 2000 to 2001	Women, (100,18–55 y)	98.0 (*95.26–100.74*)	ELISA (HerpeSelect 1, Focus Diagnostics, Cypress, CA 90630, USA); Western Blot		[114], 2004
(B) HSV-2						
Cambodia	NA					
Laos	NA					
Malaysia	Kuala Lumpur, NA	HIV-infected, (232, 32–43)	53.9 (*47.49–60.31*)	ELISA (HerpeSelect, Focus Diagnostics, Cypress, CA 90630, USA)		[69], 2017
Myanmar	NA					
Singapore	Singapore, 2003 to 2004	Sex workers, (300, 22–70 y)	79.0 (*74.39–83.61*)	ELISA (HerpeSelect 2, Focus Diagnostics, Cypress, CA 90630, USA)	Higher age; Duration of years of practice as sex worker (>9 y)	[108], 2006
	Singapore, 2003 to 2004	Attendees of STI clinic, (400, 15–80 y)	28.5 (*24.08–32.92*)	ELISA (HerpeSelect 2, Focus Diagnostics, Cypress, CA 90630, USA)		[109], 2006
Thailand Range: 14.9–80.0	Phitsanulok, 1991	Male army conscripts, (1115, 21–27)	14.9 (12.9–17.1)	Immunoblot	Higher age; Occupation (Businessmen, skilled laborers); Living area (upper North); Start of sexual activity (≤16 y); (early) sexual contact with FSW; Frequency of sexual contact with FSW (≥4 times/y)	[110,111], 1998, 1999
	Chiang Rai Province, 1991 to 1994	FSW, (500, NA)	75.6 (*71.84–79.36*)	Immunoblot	HIV status (positive)	[112], 1999
	Bangkok, 1992 to 1995	Pregnant women, (300, 14–40 y)	80.0 (*75.47–84.53*)	ELISA (Herpelisa II, Biowhittaker, USA)		[52], 1997
	Bangkok, 1996 to 1997	HIV-infected pregnant women, (307, 17–39 y)	74.3 (*69.41–79.19*)	ELISA (HerpeSelect 2, Focus Diagnostics, Cypress, CA 90630, USA)		[115], 2008
	Bangkok, 2006 to 2010	MSM, (1544, NA)	20.7 (*18.68–22.72*)	ELISA (HerpeSelect 2, Focus Diagnostics, Cypress, CA 90630, USA)	Age (≥30 y); Low level of education; Past use of drugs; Meeting casual sexual partners at a public venue; Syphilis seropositivity	[116], 2012
	Bangkok, 2006 to 2012	MSM, (1744, 18–56 y)	21.3 (*19.38–23.22*)	ELISA (HerpeSelect 2, Focus Diagnostics, Cypress, CA 90630, USA)		[113], 2013
Vietnam Range: 2.0–30.8	HCMC, 1997	Married women, (1106, 16–69 y)	30.8 (28.1–33.4)	ELISA (Focus Diagnostics, Cypress, CA)	Higher age; Low level of education; Age at first intercourse (age <19 y); Age at first pregnancy (age <21 y); Nulliparous; Number of lifetime sexual partner (>1)	[117,118], 2009, 2003
	Hanoi, 1997	Married women, (1170, 17–82 y)	8.8 (7.1–10.5)	ELISA (Focus Diagnostics, Cypress, CA)		[117,118], 2009, 2003
	Bac Ninh Province, 2003	Injection drug user, (309, 18–45 y)	22.4 (17.6–27.9)	ELISA (HerpSelect 2, MRL; Focus Technologies, Los Anglees, CA)	Resident of Bac Ninh town; Injection frequency (daily)	[119], 2006
	Lai Chau, Quang Tri, An Giang, Dong Thap, Kien Giang Province, 2002 to 2003	FSW, (904, NA)	27.7 (24.8–30.7)	ELISA (Genzyme Virotech GmbH, Russelsheim, Germany 2003)	Ethnicity (Kinh); Sex work; Number of clients (≥9/week); Ever worked outside Vietnam; >1 pregnancy termination; Syphilis seropositivity HIV status (positive)	[120], 2006
	Lai Chau, Quang Tri, An Giang, Dong Thap, Kien Giang Province, 2004	FSW, (982, NA)	24.9 (*22.2–27.6*)	NA		[121], 2007
	Hanoi, 2004	Married women, (1238, NA)	2.0 (*1.22–2.78*)	ELISA (HerpSelect 2, MRL; Focus Technologies, Los Anglees, CA), Western Blot		[122], 2008
	Hai Phong city, Do Son beach, 2007	Clients of FSW, (292, 18–60 y)	16.35 (*12.11–20.59*)	ELISA (HerpeSelect 2, Focus Diagnostics, Cypress, CA 90630, USA)	Active and potential bridgers (males with sex with FSW and lower-risk women, not using condoms)	[123], 2009
(C) Unclassified HSV						
Vietnam	Ho Chi Minh City, 1996	HIV-infected drug users, (235, 24–57 y)	99.0 (97.72–100–28)	ELISA (Behring)		[65], 1999

#### 3.6.1. Seroprevalence by Country

No studies on either HSV-1 or HSV-2 seroprevalence were identified for Cambodia, Laos, or Myanmar and only one study from Kuala Lumpur, Malaysia, reporting seroprevalence rates of 70.7% and 53.9% for HSV-1 and HSV-2, respectively, in adult HIV-infected individuals [69]. 

Two studies were identified for Singapore with seroprevalence rates of 55.8% [109] and 76.7% [108] for HSV-1, and of 28.5% [109] and 79.0% [108] for HSV-2. The studies included attendees of a sexual infection clinic that were either sex workers or general population [108,109]. In every age group of the general population, HSV-1 exceeded HSV-2 seroprevalence [109]. In contrast, in sex workers, the predominating HSV type depended on the age group [108] with HSV-2 seroprevalence exceeding HSV-1 seroprevalence in the age groups of 30 years or older. 

Seven studies were identified for Thailand and the seroprevalence for HSV-1 and HSV-2 was between 56.5% [113] to 91.0% [112] and 14.9% [110,111] to 80.0% [52], respectively. HSV-1 rates tended to be higher than HSV-2 rates, e.g., a study population of male army conscripts showed an HSV-1 seroprevalence of 77.0% and a lower HSV-2 seroprevalence of 14.9% [110,111]. The highest HSV-1 rate (91.0%) was reported among female sex workers, who had a lower HSV-2 rate (75.6%) [112]. Surprisingly, the highest HSV-2 rate of 80.0% was reported in a population of pregnant women [52]. The study cohorts were exclusively recruited in Bangkok and in the North of Thailand.

Nine studies were identified for Vietnam and the only reported HSV-1 seroprevalence was 98.0% [114] while HSV-2 seroprevalence ranged between 2.0% [122] and 30.8% [117,118]. Almost all studies covered only HSV-2 seroprevalence and in contrast to other countries, HSV-2 serosurveys from Vietnam focused especially on healthy women and sex workers. 

Overall, HSV-1 seroprevalence seemed to be similarly high in all countries, generally above 55%, irrespective of the study population. 

When comparing HSV-1 to HSV-2 seroprevalence in similar study populations, HSV-1 seroprevalence seemed to be generally higher than HSV-2 seroprevalence. Most studies (11/19) focused on high-risk groups such as sex workers and HIV-infected individuals. For HSV-1 IgG seroprevalence, there was only one study focusing on the general population [114]. For HSV-2 IgG seroprevalence, only four studies included healthy study populations [52,117,118,122]. HSV-2 IgG seroprevalence of above 50.0% was reported in HIV-infected individuals [69,115] and Thailand reported the highest HSV-2 seroprevalence overall. HSV-2 seroprevalence of sex workers in Thailand and Singapore and of HIV-infected individuals ranged between 75.6% [112] and 79.0% [108]. In contrast, sex workers in Vietnam showed a lower HSV-2 seroprevalence of 24.9% [121] to 27.7% [120]. 

One study did not report the method used [121]. Most studies (15/19) used ELISA for antibody detection and three studies used immunoblots [110,111,112]. One study used ELISA for detection and western blot as confirmation [122]. Studies using immunoblot tended to be older than studies using other methods and all recent studies used ELISA for IgG seroprevalence investigation.

#### 3.6.2. Risk Factors for Seropositivity

Most studies did not identify RFs for HSV-1 seropositivity, except for one where participants of higher age were statistically significant more likely to be seropositive [109]. 

For anti-HSV-2 IgG seropositivity, higher age was identified as statistically significant RF in Thailand, Singapore, and Vietnam in different study populations [108,111,116,117]. In line with this, one study identified age below 20 as a statistically significant RF for anti-HSV-2 IgG negativity [120]. Several studies in Thailand also identified young age at first sexual contact as statistically significant RF [111,117]. High frequency of sexual contact with female sex workers, as well as young age at first contact with female sex workers were statistically significant RFs linked to an increased anti-HSV-2 IgG seropositivity rate [111].

As mentioned, study populations of sex workers showed high HSV seroprevalence rates, regardless of the subtype [108,109]. Studies reported that sex workers were statistically significant more likely to be seropositive for HSV-2 when they already worked 9 years or longer in the sector [108] or when co-infected with other sexually transmissible diseases [112,120].

## 4. Discussion

This review provides an overview about serosurveys of ToRCH pathogens conducted in Southeast Asia during a 30-year time period. While some pathogens were well studied (e.g., *T. gondii*), only limited data were available for others, such as VZV and B19V. The majority of the studies was conducted in the better-developed countries of the region, namely Malaysia, Singapore, and Thailand. In contrast, there was a general paucity of studies from Cambodia, Laos, Myanmar and Vietnam. Moreover, seroprevalence data were often limited to a certain geographical region within the country or to certain cohorts. There was an evident lack of recent serosurveys: many studies date back to the early 2000’s or late 1990’s. Finally, the study quality (Table S1) was often poor, irrespective of the studied pathogen or the study country, but the quality of more recent articles seemed slightly better. General information such as study location and year, test method and specimen type used and overall seroprevalence result were provided by most of the studies. In contrast, quality criteria such as calculating a confidence interval, discussing bias, or presenting a sample size calculation, were met by less than one third of the studies. Well-designed and nation-wide studies on IgG seroprevalence of ToRCH pathogens are urgently needed as there are no surveillance systems for ToRCH pathogens except for RV and VZV [8,9,10]. Combined with RF analysis, these studies may lead to recommendations for infection prevention, especially during pregnancy. Studies investigating the causes of neonatal death and an analysis of the data in relation to seroprevalence information would contribute to our understanding in how far the prevalence of ToRCH pathogens and the resulting risk of infection during pregnancy influence NMR. Serosurveys about vaccine-preventable infections inform about the need to introduce a new vaccine, about the benefits and weaknesses of vaccination programs and are essential to identify target groups for SIAs. 

The *T. gondii* studies demonstrated a wide disparity in seroprevalence rates ranging from 2.6% in healthy Thai women [60] to 59.7% in the general Malay population [44]. This disparity can be explained by differing cultural or behavioral practices, as well as a wide variability regarding study design, target cohort and laboratory testing. Contact to cats, consumption of undercooked or raw meat and usage of unclean water [11,124] were identified as RFs in some studies from Thailand and Malaysia [29,39,40,53,59,61,64]. Other studies suggested high age as RF for past infection [28,29,40,41,42,46,47,61,64]. This was also observed in developed countries such as Germany [125]. The seroprevalence data found, suggest that the circulation of the parasite is higher in Malaysia, Thailand, and Myanmar than in the other countries. Women of reproductive age with the associated risky behaviors may therefore be more at risk of primary infection with the pathogen in these countries. Although preventive measures exist [126], their implementation is thwarted by knowledge gaps, which are not limited to developing countries [127,128]. Indeed, knowledge about *T. gondii* was limited among pregnant women from the Philippines, Malaysia, and Thailand [129]. While screening for *T. gondii* serostatus during antenatal care is widely discussed in the literature [12], it is so far implemented in only a few countries such as Austria and France [11]. In low resource-countries, improving the training of HCW, who can pass on crucial information during antenatal care sessions, seems more appropriate as a first approach to reduce the risk of primary infection during pregnancy.

VZV seroprevalence was rather high in all studied countries and ranged between 52.8% in young Thai adults [74] and 99.0% in middle-aged intravenous drug users in Vietnam [65]. As of October 2020, VZV vaccination is not integrated in the national immunization program of any of the studied countries [98]. However, it is recommended for susceptible HCW in Singapore and Malaysia [130,131]. Consequently, high IgG seroprevalence indicates most likely high virus circulation levels. Several studies suggested that infection occurs in late adolescence or early adulthood [71,74,75] and earlier in temperate climates [132]. Previous studies indicated that temperate weather conditions might favor virus transmission, explaining earlier immunity in these countries [133,134]. Others suggested that a tropical humid climate interferes with virus stability and that virus transmission patterns differ between the different virus variants. Supposedly because of lower population densities, virus circulation in rural settings of tropical countries is low [132]. While the high seroprevalence rates in adults suggest that the risk of primary infection during pregnancy is small, vaccination of vulnerable groups such as women of reproductive age, people with chronic diseases, children and HCW should be further discussed. 

B19V was the least studied ToRCH-pathogen in the region. The lack of recent data makes it difficult to make well-founded statements about the infection risk during pregnancy. However, the low seroprevalence ranging between 10.9% in Thai undergraduate students and 37.6% in the general Malay population [78] suggests low infection rates and therefore a limited risk of women during pregnancy. 

In contrast to B19V, many studies covered the vaccine-preventable RV infections. The main goal of rubella vaccination is the prevention of CRS [135]. However, studies showed that immunization coverage needs to be ≥ 80.0% to avoid an increased risk of CRS due to a phenomenon called paradoxical shift, when virus circulation is not interrupted and women enter reproductive age without vaccine-induced or natural immunity [135,136]. As of October 2020, all countries included in this review have introduced RCV into their routine vaccination schedule [99]. Although Singapore, Thailand, and Malaysia have provided RCV since more than 30 years [88,137,138], serosurveys suggest that more than 10% of the population remains susceptible to RV infection. Some studies from South East Asia reported seroprevalence rates below 90.0% in children [90,92,93,94], and below 85.0% in adults older than 35 years [90,91,93]. Even a more recent study from 2018 found a seroprevalence below 90.0% in some older age groups [96]. These studies indicate some vaccination gaps and suggest that some population groups were targeted neither by the national immunization programs, nor by SIAs, but the risk of infection in these countries can be considered to be low. Cambodia, Vietnam, Laos, and Myanmar introduced RCV only in 2011 or later [85,99,139,140,141,142]. Reports of the pre-vaccination era provide an estimate of virus circulation in unvaccinated populations. Here, more than 25% were susceptible to RV and only older age groups reached seroprevalence rates of more than 80% [83,97]. Infections were commonly acquired in childhood as studies reporting age-distributed data showed seroprevalence rates of 50% or above already at age 11 and older [83,84,97]. Except for a study from Laos [85], there were no studies investigating seroprevalence after RCV introduction and thus the success of the vaccination programs is largely unknown. The Lao study showed that particularly young women benefited from RCV introduction [85].

Despite the vaccination, local outbreaks of rubella have been described in recent years, which were most likely due to above mentioned immunity gaps [8,9]. Nevertheless, rubella and CRS cases have declined in Southeast Asia [8,9]. Expanding the target age groups in SIAs could be a reasonable approach to reduce the risk of infection in all women of reproductive age. Additionally, serosurveys remain important to monitor immunity gaps and to identify susceptible populations to prevent rubella outbreaks.

High levels of CMV circulation have been reported in Africa, South America, and Asia [143]. Also in Southeast Asia, high seroprevalence rates were reported throughout all study populations ranging between 52.4% in healthy adults [104] and 100.0% in pregnant women [52,106] or intravenous drug users [65]. The large majority of the studies reported high seroprevalence rates (>70%) already in young age groups, between 15 to 30 years old [49,102]. Consequently, CMV infection is also in this area frequently acquired at young age and most adults, including pregnant women, are IgG positive. However, anti-CMV antibodies do not protect from reinfection and reinfection during pregnancy can lead to congenital infection [144]. In high prevalence settings, reinfection was even considered to be responsible for most cases of congenital CMV [145,146]. Yet, a Singaporean study reported that only 20% of pregnant women had heard of CMV [147], suggesting that knowledge and awareness are low despite the high prevalence. This is also true for countries outside of the investigated region [128,148,149] and is of concern since CMV infection is considered to be the most common congenital infection worldwide [150]. Educational programs for HCW as well as for pregnant women seem to be urgently needed to reduce the risk of acquiring a CMV infection during pregnancy.

HSV-1 is another virus for which the global burden is high [151]. Seroprevalence rates in South East Asia ranged from 55.8% in 15- to 80-year-old Singaporeans [109] to 98.0% in Vietnamese 18 to 55 year old women [114]. Little age-stratified information was available suggesting acquisition of immunity during late adolescence or young adulthood since high levels of seropositivity were already found in individuals younger than 20 years [108,109]. This finding implies that women of reproductive age are largely protected from primary infection. 

Interestingly, for most countries, especially Thailand and Vietnam, more studies on HSV-2 than on HSV-1 were available, probably due to the strong influence of sex tourism in these countries. Seroprevalence of anti-HSV-2 IgG among individuals with risky sexual behavior ranged between 24.9% [121] and 79.0% [108]. Among individuals without risky sexual behavior, the seroprevalence rates were mostly below 20% but ranged between 2.0% in Vietnamese married women [122] and 80.0% in Thai pregnant women [52]. This indicates that women without risky sexual behavior are most likely not immune against HSV-2, but are also not at risk of developing genital ulcers during pregnancy due to reactivation. While transplacental transmission of HSV-1 or -2 is rather rare, mother-to-child transmission during vaginal delivery is more common [17]. RFs for seropositivity reported from South-East Asia included having multiple sexual partners, history of other sexually transmitted diseases, female sex, low educational level, belonging to minority ethnic groups and start of sexual activity at an early age [18,152]. To be able to assess virus exposure and to identify RFs, more seroprevalence surveys are needed and awareness raising campaigns should target in particular sex workers. Moreover, HSV screening should be offered during pregnancy. 

Although the present review provides a comprehensive overview of current knowledge of ToRCH pathogens in the region, there are some limitations. Geographical restrictions, different study cohorts and test methods hampered result comparability and the suboptimal quality of many studies raises concerns related to data reliability. However, despite these constraints, the review provides insights into pathogen distribution, identifies immunity gaps and susceptible populations in the region, allows a risk-estimation of primary infections during pregnancy and provides guidance for future research.

## 5. Conclusions

Women of childbearing age in Southeast Asia are susceptible to many ToRCH pathogens. The paucity of reliable information for several pathogens and the often low quality of the studies warrant comprehensive nationwide serosurveys including pregnant women but also the general population. The data could serve as a basis to evaluate and improve current prevention measures. To raise knowledge and awareness of the risks posed by ToRCH pathogens both in HCW and in pregnant women, is an important first step to prevent fetal loss and congenital malformations.

## Supplementary Materials

The following are available online at https://www.mdpi.com/2076-2607/9/3/574/s1, Table S1: Assessment of the quality of the studies included in this review.

## References

[1] Coyne C.B., Lazear H.M. (2016). Zika virus-reigniting the TORCH. Nat. Rev. Microbiol..

[2] Schwartz D.A. (2017). The Origins and Emergence of Zika Virus, the Newest TORCH Infection: What’s Old Is New Again. Arch. Pathol. Lab Med..

[3] Adams Waldorf K.M., McAdams R.M. (2013). Influence of infection during pregnancy on fetal development. Reproduction.

[4] Madrid L., Varo R., Sitoe A., Bassat Q. (2016). Congenital and perinatally-acquired infections in resource-constrained settings. Expert Rev. Anti Infect. Ther..

[5] Neu N., Duchon J., Zachariah P. (2015). TORCH infections. Clin. Perinatol..

[6] Menson E., Lyall H. (2005). Clinical presentation of congenital viral infection. Curr. Paediatr..

[7] Vynnycky E., Adams E.J., Cutts F.T., Reef S.E., Navar A.M., Simons E., Yoshida L.M., Brown D.W., Jackson C., Strebel P.M. (2016). Using Seroprevalence and Immunisation Coverage Data to Estimate the Global Burden of Congenital Rubella Syndrome, 1996-2010: A Systematic Review. PLoS ONE.

[8] Khanal S., Bahl S., Sharifuzzaman M., Dhongde D., Pattamadilok S., Reef S., Morales M., Dabbagh A., Kretsinger K., Patel M. (2018). Progress Toward Rubella and Congenital Rubella Syndrome Control-South-East Asia Region, 2000–2016. MMWR Morb. Mortal Wkly. Rep..

[9] Knapp J.K., Mariano K.M., Pastore R., Grabovac V., Takashima Y., Alexander J.P., Reef S.E., Hagan J.E. (2020). Progress Toward Rubella Elimination-Western Pacific Region, 2000–2019. MMWR Morb. Mortal Wkly. Rep..

[10] World Health Organization (2018). JRF Supplementary Questionnaire on Surveillance 2017.

[11] Montoya J.G., Liesenfeld O. (2004). Toxoplasmosis. Lancet.

[12] Wallon M., Peyron F. (2018). Congenital Toxoplasmosis: A Plea for a Neglected Disease. Pathogens.

[13] Sauerbrei A., Wutzler P. (2001). Neonatal varicella. J. Perinatol..

[14] Sauerbrei A., Wutzler P. (2007). Herpes simplex and varicella-zoster virus infections during pregnancy: Current concepts of prevention, diagnosis and therapy. Part 2: Varicella-zoster virus infections. Med. Microbiol. Immunol..

[15] Dontigny L., Arsenault M.Y., Martel M.J., Clinical Practice Obstetrics Committee (2008). Rubella in pregnancy. J. Obstet. Gynaecol. Can..

[16] Akpan U.S., Pillarisetty L.S. Congenital Cytomegalovirus Infection (Congenital CMV Infection). https://www.ncbi.nlm.nih.gov/books/NBK541003/.

[17] Sauerbrei A., Wutzler P. (2007). Herpes simplex and varicella-zoster virus infections during pregnancy: Current concepts of prevention, diagnosis and therapy. Part 1: Herpes simplex virus infections. Med. Microbiol. Immunol..

[18] Bhatta A.K., Keyal U., Liu Y., Gellen E. (2018). Vertical transmission of herpes simplex virus: An update. J. Dtsch. Derm. Ges.

[19] UNICEF, United Nations Inter-Agency Group for Child Mortality Estimation (UN IGME) (2019). Child Mortality Estimates Country-Specific Neonatal Mortality Rate.

[20] United Nations Inter-Agency Group for Child Mortality Estimation (UN IGME) (2019). *Levels & Trends in Child Mortality: Report 2019*; United Nations Inter-Agency Group for Child Mortality Estimation. https://www.unicef.org/media/60561/file/UN-IGME-child-mortality-report-2019.pdf.

[21] Cutts F.T., Hanson M. (2016). Seroepidemiology: An underused tool for designing and monitoring vaccination programmes in low- and middle-income countries. Trop. Med. Int. Health.

[22] Thompson K.M., Odahowski C.L. (2016). Systematic Review of Measles and Rubella Serology Studies. Risk Anal. Off. Publ. Soc. Risk Anal..

[23] Creative Research Systems The Survey System. https://www.surveysystem.com/sscalc.htm.

[24] Dimech W., Mulders M.N. (2016). A 16-year review of seroprevalence studies on measles and rubella. Vaccine.

[25] Moher D., Liberati A., Tetzlaff J., Altman D. (2009). Preferred Reporting Items for Systematic Reviews and Meta-Analyses: The PRISMA Statement. PLoS Med..

[26] Richard-Lenoble D., Cheng H.K., Sire J.M., Duong T.H., Cheng T.V., Phanny I., Rainsy T., Cauchoix C. (1999). Toxoplasmosis in Cambodia: Initial serological evaluation at Phnom Penh. Sante.

[27] Priest J.W., Jenks M.H., Moss D.M., Mao B., Buth S., Wannemuehler K., Soeung S.C., Lucchi N.W., Udhayakumar V., Gregory C.J. (2016). Integration of Multiplex Bead Assays for Parasitic Diseases into a National, Population-Based Serosurvey of Women 15-39 Years of Age in Cambodia. PLoS Negl. Trop. Dis..

[28] Catar G., Giboda M., Gutvirth J., Hongvanthong B. (1992). Seroepidemiological study of toxoplasmosis in Laos. Southeast Asian J. Trop. Med. Public Health.

[29] Andiappan H., Nissapatorn V., Sawangjaroen N., Nyunt M.H., Lau Y.L., Khaing S.L., Aye K.M., Mon N.C., Tan T.C., Kumar T. (2014). Comparative study on Toxoplasma infection between Malaysian and Myanmar pregnant women. Parasites Vectors.

[30] Hakim S.L., Radzan T., Nazma M. (1994). Distribution of anti-Toxoplasma gondii antibodies among Orang Asli (aborigines) in Peninsular Malaysia. Southeast Asian J. Trop. Med. Public Health.

[31] Nissapatorn V., Kamarulzaman A., Init I., Tan L.H., Rohela M., Norliza A., Chan L.L., Latt H.M., Anuar A.K., Quek K.F. (2002). Seroepidemiology of toxoplasmosis among HIV-infected patients and healthy blood donors. Med. J. Malays..

[32] Nissapatorn V., Lee C.K., Khairul A.A. (2003). Seroprevalence of toxoplasmosis among AIDS patients in Hospital Kuala Lumpur, 2001. Singap. Med. J..

[33] Nissapatorn V., Lee C., Quek K.F., Leong C.L., Mahmud R., Abdullah K.A. (2004). Toxoplasmosis in HIV/AIDS patients: A current situation. Jpn. J. Infect. Dis..

[34] Nissapatorn V., Lee C.K., Cho S.M., Rohela M., Anuar A.K., Quek K.F., Latt H.M. (2003). Toxoplasmosis in HIV/AIDS patients in Malaysia. Southeast Asian J. Trop. Med. Public Health.

[35] Nissapatorn V., Noor Azmi M.A., Cho S.M., Fong M.Y., Init I., Rohela M., Khairul Anuar A., Quek K.F., Latt H.M. (2003). Toxoplasmosis: Prevalence and risk factors. J. Obstet. Gynaecol. J. Inst. Obstet. Gynaecol..

[36] Nissapatorn V., Lim Y.A., Jamaiah I., Agnes L.S., Amyliana K., Wen C.C., Nurul H., Nizam S., Quake C.T., Valartmathi C. (2005). Parasitic infections in Malaysia: Changing and challenges. Southeast Asian J. Trop. Med. Public Health.

[37] Chan B.T., Amal R.N., Hayati M.I., Kino H., Anisah N., Norhayati M., Sulaiman O., Abdullah M.M., Fatmah M.S., Roslida A.R. (2008). Seroprevalence of toxoplasmosis among migrant workers from different Asian countries working in Malaysia. Southeast Asian J. Trop. Med. Public Health.

[38] Nissapatorn V., Leong T.H., Lee R., Init I., Ibrahim J., Yen T.S. (2011). Seroepidemiology of toxoplasmosis in renal patients. Southeast Asian J. Trop. Med. Public Health.

[39] Nimir A., Othman A., Ee S., Musa Z., Majid I.A., Kamarudin Z., Xian C., Isa N.H. (2010). Latent toxoplasmosis in patients with different malignancy: A hospital based study. J. Clin. Med. Res..

[40] Ngui R., Lim Y.A., Amir N.F., Nissapatorn V., Mahmud R. (2011). Seroprevalence and sources of toxoplasmosis among Orang Asli (indigenous) communities in Peninsular Malaysia. Am. J. Trop. Med. Hyg..

[41] Singh S., Khang T.F., Andiappan H., Nissapatorn V., Subrayan V. (2012). An age-adjusted seroprevalence study of Toxoplasma antibody in a Malaysian ophthalmology unit. Trans. R. Soc. Trop. Med. Hyg..

[42] Emelia O., Amal R.N., Ruzanna Z.Z., Shahida H., Azzubair Z., Tan K.S., Noor Aadila S., Siti N.A., Aisah M.Y. (2012). Seroprevalence of anti-Toxoplasma gondii IgG antibody in patients with schizophrenia. Trop. Biomed..

[43] Omar A., Bakar O.C., Adam N.F., Osman H., Osman A., Suleiman A.H., Manaf M.R., Selamat M.I. (2015). Seropositivity and serointensity of Toxoplasma gondii antibodies and DNA among patients with schizophrenia. Korean J. Parasitol..

[44] Ahmad A.F., Ngui R., Muhammad Aidil R., Lim Y.A., Rohela M. (2014). Current status of parasitic infections among Pangkor Island community in Peninsular Malaysia. Trop. Biomed..

[45] Emelia O., Rahana A.R., Mohamad Firdaus A., Cheng H.S., Nursyairah M.S., Fatinah A.S., Azmawati M.N., Siti N.A., Aisah M.Y. (2014). IgG avidity assay: A tool for excluding acute toxoplasmosis in prolonged IgM titer sera from pregnant women. Trop. Biomed..

[46] Angal L., Lim Y.A., Yap N.J., Ngui R., Amir A., Kamarulzaman A., Rohela M. (2016). Toxoplasmosis in HIV and non HIV prisoners in Malaysia. Trop. Biomed..

[47] Brandon-Mong G.J., Che Mat Seri N.A., Sharma R.S., Andiappan H., Tan T.C., Lim Y.A., Nissapatorn V. (2015). Seroepidemiology of Toxoplasmosis among People Having Close Contact with Animals. Front. Immunol..

[48] van Enter B.J.D., Lau Y.L., Ling C.L., Watthanaworawit W., Sukthana Y., Lee W.C., Nosten F., McGready R. (2017). Seroprevalence of Toxoplasma gondii Infection in Refugee and Migrant Pregnant Women along the Thailand-Myanmar Border. Am. J. Trop. Med. Hyg..

[49] Wong A., Tan K.H., Tee C.S., Yeo G.S. (2000). Seroprevalence of cytomegalovirus, toxoplasma and parvovirus in pregnancy. Singap. Med. J..

[50] Lim R.B., Tan M.T., Young B., Lee C.C., Leo Y.S., Chua A., Ng O.T. (2013). Risk factors and time-trends of cytomegalovirus (CMV), syphilis, toxoplasmosis and viral hepatitis infection and seroprevalence in human immunodeficiency virus (HIV) infected patients. Ann. Acad. Med. Singap..

[51] Chintana T. (1991). Pattern of antibodies in toxoplasmosis of pregnant women and their children in Thailand. Southeast Asian J. Trop. Med. Public Health.

[52] Taechowisan T., Sutthent R., Louisirirotchanakul S., Puthavathana P., Wasi C. (1997). Immune status in congenital infections by TORCH agents in pregnant Thais. Asian Pac. J. Allergy Immunol..

[53] Chintana T., Sukthana Y., Bunyakai B., Lekkla A. (1998). Toxoplasma gondii antibody in pregnant women with and without HIV infection. Southeast Asian J. Trop. Med. Public Health.

[54] Sukthana Y. (1999). Difference of Toxoplasma gondii antibodies between Thai and Austrian pregnant women. Southeast Asian J. Trop. Med. Public Health.

[55] Maruyama S., Boonmar S., Morita Y., Sakai T., Tanaka S., Yamaguchi F., Kabeya H., Katsube Y. (2000). Seroprevalence of Bartonella henselae and Toxoplasma gondii among healthy individuals in Thailand. J. Vet. Med. Sci..

[56] Pinlaor S., Ieamviteevanich K., Pinlaor P., Maleewong W., Pipitgool V. (2000). Seroprevalence of specific total immunoglobulin (Ig), IgG and IgM antibodies to Toxoplasma gondii in blood donors from Loei Province, Northeast Thailand. Southeast Asian J. Trop. Med. Public Health.

[57] Wanachiwanawin D., Sutthent R., Chokephaibulkit K., Mahakittikun V., Ongrotchanakun J., Monkong N. (2001). Toxoplasma gondii antibodies in HIV and non-HIV infected Thai pregnant women. Asian Pac. J. Allergy Immunol..

[58] Tantivanich S., Amarapal P., Suphadtanaphongs W., Siripanth C., Sawatmongkonkun W. (2001). Prevalence of congenital cytomegalovirus and Toxoplasma antibodies in Thailand. Southeast Asian J. Trop. Med. Public Health.

[59] Sukthana Y., Kaewkungwal J., Jantanavivat C., Lekkla A., Chiabchalard R., Aumarm W. (2003). Toxoplasma gondii antibody in Thai cats and their owners. Southeast Asian J. Trop. Med. Public Health.

[60] Sakae C., Natphopsuk S., Settheetham-Ishida W., Ishida T. (2013). Low prevalence of Toxoplasma gondii infection among women in northeastern Thailand. J. Parasitol..

[61] Nissapatorn V., Suwanrath C., Sawangjaroen N., Ling L.Y., Chandeying V. (2011). Toxoplasmosis-serological evidence and associated risk factors among pregnant women in southern Thailand. Am. J. Trop. Med. Hyg..

[62] Chemoh W., Sawangjaroen N., Nissapatorn V., Suwanrath C., Chandeying V., Hortiwakul T., Andiappan H., Sermwittayawong N., Charoenmak B., Siripaitoon P. (2013). Toxoplasma gondii infection: What is the real situation?. Exp. Parasitol..

[63] Chemoh W., Sawangjaroen N., Siripaitoon P., Andiappan H., Hortiwakul T., Sermwittayawong N., Charoenmak B., Nissapatorn V. (2015). Toxoplasma gondii-Prevalence and Risk Factors in HIV-infected Patients from Songklanagarind Hospital, Southern Thailand. Front. Microbiol..

[64] Andiappan H., Nissapatorn V., Sawangjaroen N., Chemoh W., Lau Y.L., Kumar T., Onichandran S., Suwanrath C., Chandeying V. (2014). Toxoplasma infection in pregnant women: A current status in Songklanagarind hospital, southern Thailand. Parasites Vectors.

[65] Follezou J.Y., Lan N.Y., Lien T.X., Lafon M.E., Tram L.T., Hung P.V., Aknine X., Lowenstein W., Ngai N.V., Theodorou I. (1999). Clinical and biological characteristics of human immunodeficiency virus-infected and uninfected intravascular drug users in Ho Chi Minh City, Vietnam. Am. J. Trop. Med. Hyg..

[66] Udonsom R., Lekkla A., Chung P.T., Cam P.D., Sukthana Y. (2008). Seroprevalence of Toxoplasma gondii antibody in Vietnamese villagers. Southeast Asian J. Trop. Med. Public Health.

[67] Buchy P., Follezou J.Y., Lien T.X., An T.T., Tram L.T., Tri D.V., Cuong N.M., Glaziou P., Chien B.T. (2003). Serological study of toxoplasmosis in Vietnam in a population of drug users (Ho Chi Minh city) and pregnant women (Nha Trang). Bull. De La Soc. De Pathol. Exot..

[68] Black A.P., Vilivong K., Nouanthong P., Souvannaso C., Hubschen J.M., Muller C.P. (2015). Serosurveillance of vaccine preventable diseases and hepatitis C in healthcare workers from Lao PDR. PLoS ONE.

[69] Yap S.H., Abdullah N.K., McStea M., Takayama K., Chong M.L., Crisci E., Larsson M., Azwa I., Kamarulzaman A., Leong K.H. (2017). HIV/Human herpesvirus co-infections: Impact on tryptophan-kynurenine pathway and immune reconstitution. PLoS ONE.

[70] Dashraath P., Ong E.S., Lee V.J. (2007). Seroepidemiology of varicella and the reliability of a self-reported history of varicella infection in Singapore military recruits. Ann. Acad. Med. Singap..

[71] Fatha N., Ang L.W., Goh K.T. (2014). Changing seroprevalence of varicella zoster virus infection in a tropical city state, Singapore. Int. J. Infect. Dis. IJID Off. Publ. Int. Soc. Infect. Dis..

[72] Gorny A.W., Mittal C., Saw S., Venkatachalam I., Fisher D.A., Tambyah P.A. (2015). Varicella seroprevalence in healthcare workers in a tertiary hospital: An audit of cross-sectional data. BMC Res. Notes.

[73] Migasena S., Simasathien S., Desakorn V., Phonrat B., Suntharasamai P., Pitisuttitham P., Aree C., Naksrisook S., Supeeranun L., Samakoses R. (1997). Seroprevalence of Varicella-Zoster Virus Antibody in Thailand. Int. J. Infect. Dis. IJID Off. Publ. Int. Soc. Infect. Dis..

[74] Lolekha S., Tanthiphabha W., Sornchai P., Kosuwan P., Sutra S., Warachit B., Chup-Upprakarn S., Hutagalung Y., Weil J., Bock H.L. (2001). Effect of climatic factors and population density on varicella zoster virus epidemiology within a tropical country. Am. J. Trop. Med. Hyg..

[75] Kowitdamrong E., Pancharoen C., Thammaborvorn R., Bhattarakosol P. (2005). The prevalence of varicella-zoster virus infection in normal healthy individuals aged above 6 months. J. Med Assoc. Thail. Chotmaihet Thangphaet.

[76] Srichomkwun P., Apisarnthanarak A., Thongphubeth K., Yuekyen C., Mundy L.M. (2009). Evidence of vaccine protection among thai medical students and implications for occupational health. Infect. Control Hosp. Epidemiol..

[77] Suwanpakdee D., Laohapand C., Moolasart V., Lomtong P., Krairojananan N., Srisawat P., Watanaveeradej V. (2012). Serosurveillance of varicella and hepatitis B infection after reported cases in medical students and the relationship between past varicella disease history and immunity status. J. Med. Assoc. Thail. Chotmaihet Thangphaet.

[78] Ooi S.L., Hooi P.S., Chua B.H., Lam S.K., Chua K.B. (2002). Seroprevalence of human parvovirus B19 infection in an urban population in Malaysia. Med. J. Malays..

[79] Matsunaga Y., Goh K.T., Utagawa E., Muroi N. (1994). Low prevalence of antibody to human parvovirus B19 in Singapore. Epidemiol. Infect..

[80] Poovorawan Y., Theamboonlers A., Suandork P., Hirsch P. (2000). Prevalence of antibodies to parvovirus B 19 in Thailand. Southeast Asian J. Trop. Med. Public Health.

[81] Suandork P., Theamboonlers A., Likitnukul S., Hirsch P., Poovorawan Y. (2000). Parvovirus B19 antibodies in immunocompromized children in Thailand. Asian Pac. J. Allergy Immunol..

[82] Bhattarakosol P., Pancharoen C., Kowitdamrong E., Thammaborvorn R., Mungmee V. (2003). Prevalence of parvovirus B19 infection in Thai young adults. Southeast Asian J. Trop. Med. Public Health.

[83] Mao B., Chheng K., Wannemuehler K., Vynnycky E., Buth S., Soeung S.C., Reef S., Weldon W., Quick L., Gregory C.J. (2015). Immunity to polio, measles and rubella in women of child-bearing age and estimated congenital rubella syndrome incidence, Cambodia, 2012. Epidemiol. Infect..

[84] Phengxay M., Hayakawa Y., Phan T.G., Uneno-Yamamoto K., Tanaka-Taya K., Vongphrachanh P., Komase K., Ushijima H. (2011). Seroprevalence of rubella and measles antibodies in Lao PDR. Clin. Lab..

[85] Hachiya M., Miyano S., Mori Y., Vynnycky E., Keungsaneth P., Vongphrachanh P., Xeuatvongsa A., Sisouk T., Som-Oulay V., Khamphaphongphane B. (2018). Evaluation of nationwide supplementary immunization in Lao People’s Democratic Republic: Population-based seroprevalence survey of anti-measles and anti-rubella IgG in children and adults, mathematical modelling and a stability testing of the vaccine. PLoS ONE.

[86] Sekawi Z., Muizatul W.M., Marlyn M., Jamil M.A., Ilina I. (2005). Rubella vaccination programme in Malaysia: Analysis of a seroprevalence study in an antenatal clinic. Med. J. Malays..

[87] Cheong A.T., Khoo E.M. (2008). Prevalence of rubella susceptibility among pregnant mothers in a community-based antenatal clinic in Malaysia: A cross-sectional study. Asia Pac. J. Public Health.

[88] Cheong A.T., Tong S.F., Khoo E.M. (2013). How useful is a history of rubella vaccination for determination of disease susceptibility? A cross-sectional study at a public funded health clinic in Malaysia. BMC Fam. Pract..

[89] Ang L.W., Chua L.T., James L., Goh K.T. (2010). Epidemiological surveillance and control of rubella in Singapore, 1991–2007. Ann. Acad. Med. Singap..

[90] Liew F., Ang L.W., Cutter J., James L., Goh K.T. (2010). Evaluation on the effectiveness of the national childhood immunisation programme in Singapore, 1982–2007. Ann. Acad. Med. Singap..

[91] Chua Y.X., Ang L.W., Low C., James L., Cutter J.L., Goh K.T. (2015). An epidemiological assessment towards elimination of rubella and congenital rubella syndrome in Singapore. Vaccine.

[92] Ang L.W., Lai F.Y., Tey S.H., Cutter J., James L., Goh K.T. (2013). Prevalence of antibodies against measles, mumps and rubella in the childhood population in Singapore, 2008–2010. Epidemiol. Infect..

[93] Boonruang S., Buppasiri P. (2005). Rubella antibodies in normal pregnant women at Srinagarind Hospital, Khon Kaen, Thailand. J. Med. Assoc. Thail. Chotmaihet Thangphaet.

[94] Tharmaphornpilas P., Yoocharean P., Rasdjarmrearnsook A.O., Theamboonlers A., Poovorawan Y. (2009). Seroprevalence of antibodies to measles, mumps, and rubella among Thai population: Evaluation of measles/MMR immunization programme. J. Health Popul. Nutr..

[95] Chaiwarith R., Praparattanapan J., Nuket K., Kotarathitithum W., Supparatpinyo K. (2016). Seroprevalence of antibodies to measles, mumps, and rubella, and serologic responses after vaccination among human immunodeficiency virus (HIV)-1 infected adults in Northern Thailand. BMC Infect. Dis..

[96] Sreepian P.M., Sreepian A. (2018). Seroprevalence of Rubella Immunity among women of childbearing age in Bangkok, Thailand. Southeast Asian J. Trop. Med. Public Health.

[97] Miyakawa M., Yoshino H., Yoshida L.M., Vynnycky E., Motomura H., Tho le H., Thiem V.D., Ariyoshi K., Anh D.D., Moriuchi H. (2014). Seroprevalence of rubella in the cord blood of pregnant women and congenital rubella incidence in Nha Trang, Vietnam. Vaccine.

[98] World Health Organization WHO Vaccine-Preventable Diseases: Monitoring System. 2020 Global Summary. https://apps.who.int/immunization_monitoring/globalsummary/schedules.

[99] World Health Organization (2018). Vaccine Introduction.

[100] Ahmed S.A., Al-Joudi F.S., Zaidah A.W., Roshan T.M., Rapiaah M., Abdullah Y.M., Rosline H. (2006). The prevalence of human cytomegalovirus seropositivity among blood donors at the Unit of Blood Transfusion Medicine, Hospital Universiti Sains Malaysia. Southeast Asian J. Trop. Med. Public Health.

[101] Saraswathy T.S., Az-Ulhusna A., Asshikin R.N., Suriani S., Zainah S. (2011). Seroprevalence of cytomegalovirus infection in pregnant women and associated role in obstetric complications: A preliminary study. Southeast Asian J. Trop. Med. Public Health.

[102] Urwijitaroon Y., Teawpatanataworn S., Kitjareontarm A. (1993). Prevalence of cytomegalovirus antibody in Thai-northeastern blood donors. Southeast Asian J. Trop. Med. Public Health.

[103] Tantivanich S., Suphadtanaphongs V., Siripanth C., Desakorn V., Suphanit I., Phromin S., Panakitsuwan S., Amarapand P. (1999). Prevalence of cytomegalovirus antibodies among various age groups of Thai population. Southeast Asian J. Trop. Med. Public Health.

[104] Amarapal P., Tantivanich S., Balachandra K. (2001). Prevalence of cytomegalovirus in Thai blood donors by monoclonal staining of blood leukocytes. Southeast Asian J. Trop. Med. Public Health.

[105] Fongsarun J.E., Ekkapongpisit M., Paisan M., Chanthachorn S., Papadopoulos K.I. (2013). Prevalence of transmissible viral disease in maternal blood samples of autologous umbilical cord blood in a private cord blood bank. Transplant. Technol..

[106] Bhattarakosol P., Sithidajporn M., Bhattarakosol P. (1998). Seroprevalence of cytomegalovirus infection in Thai adults detecting by ELISA. Chula Med. J..

[107] O’Charoen, Nuchprayoon C., Chumnijarakij T., Ganapi S. Cytomegalovirus Antibody Screening Program of Thai Blood Donors for Bone-Marrow Transplant Patients. http://www.tsh.or.th/file_upload/files/v2%20n1%20023.pdf.

[108] Theng T.S., Sen P.R., Tan H.H., Wong M.L., Chan K.W. (2006). Seroprevalence of HSV-1 and 2 among sex workers attending a sexually transmitted infection clinic in Singapore. Int. J. STD AIDS.

[109] Theng C.T., Sen P.R., Chio T.W., Tan H.H., Wong M.L., Chan R.K. (2006). Seroprevalence of herpes simplex virus-1 and -2 in attendees of a sexually transmitted infection clinic in Singapore. Sex. Health.

[110] Nopkesorn T., Mock P.A., Mastro T.D., Sangkharomya S., Sweat M., Limpakarnjanarat K., Laosakkitiboran J., Young N.L., Morse S.A., Schmid S. (1998). HIV-1 subtype E incidence and sexually transmitted diseases in a cohort of military conscripts in northern Thailand. J. Acquir. Immune Defic. Syndr. Hum. Retrovirol. Off. Publ. Int. Retrovirol. Assoc..

[111] Dobbins J.G., Mastro T.D., Nopkesorn T., Sangkharomya S., Limpakarnjanarat K., Weniger B.G., Schmid D.S. (1999). Herpes in the time of AIDS: A comparison of the epidemiology of HIV-1 and HSV-2 in young men in northern Thailand. Sex. Transm. Dis..

[112] Limpakarnjanarat K., Mastro T.D., Saisorn S., Uthaivoravit W., Kaewkungwal J., Korattana S., Young N.L., Morse S.A., Schmid D.S., Weniger B.G. (1999). HIV-1 and other sexually transmitted infections in a cohort of female sex workers in Chiang Rai, Thailand. Sex. Transm. Infect..

[113] van Griensven F., Thienkrua W., McNicholl J., Wimonsate W., Chaikummao S., Chonwattana W., Varangrat A., Sirivongrangson P., Mock P.A., Akarasewi P. (2013). Evidence of an explosive epidemic of HIV infection in a cohort of men who have sex with men in Thailand. Aids.

[114] Ashley-Morrow R., Nollkamper J., Robinson N.J., Bishop N., Smith J. (2004). Performance of focus ELISA tests for herpes simplex virus type 1 (HSV-1) and HSV-2 antibodies among women in ten diverse geographical locations. Clin. Microbiol. Infect. Off. Publ. Eur. Soc. Clin. Microbiol. Infect. Dis..

[115] Bollen L.J., Whitehead S.J., Mock P.A., Leelawiwat W., Asavapiriyanont S., Chalermchockchareonkit A., Vanprapar N., Chotpitayasunondh T., McNicholl J.M., Tappero J.W. (2008). Maternal herpes simplex virus type 2 coinfection increases the risk of perinatal HIV transmission: Possibility to further decrease transmission?. Aids.

[116] Holtz T.H., Thienkrua W., McNicholl J.M., Wimonsate W., Chaikummao S., Chonwattana W., Wasinrapee P., Varangrat A., Mock P.A., Sirivongrangson P. (2012). Prevalence of Treponema pallidum seropositivity and herpes simplex virus type 2 infection in a cohort of men who have sex with men, Bangkok, Thailand, 2006–2010. Int. J. STD AIDS.

[117] Le H.V., Schoenbach V.J., Herrero R., Hoang Pham A.T., Nguyen H.T., Nguyen T.T., Munoz N., Franceschi S., Vaccarella S., Parkin M.D. (2009). Herpes simplex virus type-2 seropositivity among ever married women in South and north Vietnam: A population-based study. Sex. Transm. Dis..

[118] Anh P.T.H., Hieu N.T., Herrero R., Vaccarella S., Smith J.S., Nguyen Thuy T.T., Nguyen H.N., Nguyen B.D., Ashley R., Snijders P.J. (2003). Human papillomavirus infection among women in South and North Vietnam. Int. J. Cancer.

[119] Go V.F., Frangakis C., Nam le V., Bergenstrom A., Sripaipan T., Zenilman J.M., Celentano D.D., Quan V.M. (2006). High HIV sexual risk behaviors and sexually transmitted disease prevalence among injection drug users in Northern Vietnam: Implications for a generalized HIV epidemic. J. Acquir. Immune Defic. Syndr..

[120] O’Farrell N., Thuong N.V., Nghia K.V., Tram L.T., Long N.T. (2006). HSV-2 antibodies in female sex workers in Vietnam. Int. J. STD AIDS.

[121] Thuong N.V., Van Nghia K., Hau T.P., Long N.T., Van C.T., Duc B.H., Tram L.T., Tuan N.A., Tien N.T., Godwin P. (2007). Impact of a community sexually transmitted infection/HIV intervention project on female sex workers in five border provinces of Vietnam. Sex. Transm. Infect..

[122] Ngo T.D., Laeyendecker O., La H., Hogrefe W., Morrow R.A., Quinn T.C. (2008). Use of commercial enzyme immunoassays to detect antibodies to the herpes simplex virus type 2 glycoprotein G in a low-risk population in Hanoi, Vietnam. Clin. Vaccine Immunol. CVI.

[123] Nguyen N.T., Nguyen H.T., Trinh H.Q., Mills S.J., Detels R. (2009). Clients of female sex workers as a bridging population in Vietnam. AIDS Behav..

[124] Hill D., Dubey J.P. (2002). Toxoplasma gondii: Transmission, diagnosis and prevention. Clin. Microbiol. Infect. Off. Publ. Eur. Soc. Clin. Microbiol. Infect. Dis..

[125] Pleyer U., Gross U., Schluter D., Wilking H., Seeber F. (2019). Toxoplasmosis in Germany. Dtsch. Arztebl. Int..

[126] Centers for Disease Control and Prevention Parasites-Toxoplasmosis (Toxoplasma Infection) Prevention & Control. https://www.cdc.gov/parasites/toxoplasmosis/prevent.html.

[127] Jones J.L., Ogunmodede F., Scheftel J., Kirkland E., Lopez A., Schulkin J., Lynfield R. (2003). Toxoplasmosis-related knowledge and practices among pregnant women in the United States. Infect Dis Obs. Gynecol.

[128] Pereboom M.T., Mannien J., Spelten E.R., Schellevis F.G., Hutton E.K. (2013). Observational study to assess pregnant women’s knowledge and behaviour to prevent toxoplasmosis, listeriosis and cytomegalovirus. BMC Pregnancy Childbirth.

[129] Andiappan H., Nissapatorn V., Sawangjaroen N., Khaing S.L., Salibay C.C., Cheung M.M., Dungca J.Z., Chemoh W., Xiao Teng C., Lau Y.L. (2014). Knowledge and practice on Toxoplasma infection in pregnant women from Malaysia, Philippines, and Thailand. Front. Microbiol..

[130] Mun San L. (2015). Vaccination for HCWs. SMA News.

[131] Ministry of Health Malaysia (2003). Adult Vaccination. https://de.slideshare.net/Rubzzzz/malaysia-cpg-for-adult-vaccination.

[132] Daulagala S., Noordeen F. (2018). Epidemiology and factors influencing varicella infections in tropical countries including Sri Lanka. Virusdisease.

[133] Lee B.W. (1998). Review of varicella zoster seroepidemiology in India and Southeast Asia. Trop. Med. Int. Health.

[134] World Health Organization (2014). Varicella and herpes zoster vaccines: WHO position paper, June 2014. Wkly. Epidemiol. Rec..

[135] World Health Organization (2011). Rubella vaccines: WHO position paper. Wkly. Epidemiol. Rec..

[136] (2017). Regional Strategy and Plan of Action for Measles and Rubella Elimination in the Western Pacific.

[137] Singapore Ministry of Health (2006). Congenital rubella prevention in Singapore: A success story. Epidemiol. News Bull..

[138] World Health Organization (Old Version) (2016). EPI Fact Sheet Thailand. World Health Organization. New Dheli, Most Recent Version. https://apps.who.int/iris/handle/10665/336764?locale-attribute=de&.

[139] Centers for Disease Control and Prevention Success in Cambodia: The Disappearance of Measles! (With Rubella Not Far Behind). https://www.cdc.gov/globalhealth/immunization/stories/success-in-cambodia.htm.

[140] World Health Organization (Old Version) (2018). Summary of Supplementary Immunization Activities from 2000 to 2019. World Health Organization. Most Recent Version. https://www.who.int/teams/immunization-vaccines-and-biologicals/immunization-analysis-and-insights/global-monitoring/data-statistics-and-graphics.

[141] World Health Organization (Old Version) (2016). EPI Fact Sheet Myanmar. World Health Organization. Most Recent Version. https://apps.who.int/iris/handle/10665/329987.

[142] Vynnycky E., Yoshida L.M., Huyen D.T., Trung N.D., Toda K., Cuong N.V., Thi Hong D., Ariyoshi K., Miyakawa M., Moriuchi H. (2016). Modeling the impact of rubella vaccination in Vietnam. Hum. Vaccines Immunother..

[143] Cannon M.J., Schmid D.S., Hyde T.B. (2010). Review of cytomegalovirus seroprevalence and demographic characteristics associated with infection. Rev. Med. Virol..

[144] Manicklal S., Emery V.C., Lazzarotto T., Boppana S.B., Gupta R.K. (2013). The “silent” global burden of congenital cytomegalovirus. Clin. Microbiol. Rev..

[145] Lanzieri T.M., Dollard S.C., Bialek S.R., Grosse S.D. (2014). Systematic review of the birth prevalence of congenital cytomegalovirus infection in developing countries. Int. J. Infect. Dis. IJID Off. Publ. Int. Soc. Infect. Dis..

[146] de Vries J.J., van Zwet E.W., Dekker F.W., Kroes A.C., Verkerk P.H., Vossen A.C. (2013). The apparent paradox of maternal seropositivity as a risk factor for congenital cytomegalovirus infection: A population-based prediction model. Rev. Med. Virol..

[147] Lim S.L., Tan W.C., Tan L.K. (2012). Awareness of and attitudes toward congenital cytomegalovirus infection among pregnant women in Singapore. Int. J. Gynaecol. Obstet. Off. Organ Int. Fed. Gynaecol. Obstet..

[148] Jeon J., Victor M., Adler S.P., Arwady A., Demmler G., Fowler K., Goldfarb J., Keyserling H., Massoudi M., Richards K. (2006). Knowledge and awareness of congenital cytomegalovirus among women. Infect. Dis. Obs. Gynecol..

[149] Ross D.S., Victor M., Sumartojo E., Cannon M.J. (2008). Women’s knowledge of congenital cytomegalovirus: Results from the 2005 HealthStyles survey. J. Womens Health (Larchmt.).

[150] Revello M.G., Tibaldi C., Masuelli G., Frisina V., Sacchi A., Furione M., Arossa A., Spinillo A., Klersy C., Ceccarelli M. (2015). Prevention of Primary Cytomegalovirus Infection in Pregnancy. EBioMedicine.

[151] Looker K.J., Garnett G.P., Schmid G.P. (2008). An estimate of the global prevalence and incidence of herpes simplex virus type 2 infection. Bull. World Health Organ..

[152] Smith J.S., Robinson N.J. (2002). Age-specific prevalence of infection with herpes simplex virus types 2 and 1: A global review. J. Infect. Dis..

