# Supplementary material for: Seroprevalence of ToRCH Pathogens in Southeast Asia
Source: Microorganisms. 2021 Mar 11;9(3):574. doi: 10.3390/microorganisms9030574 (PMC7999562; doi:10.3390/microorganisms9030574)
Supplement: Supplementary file 1 [file microorganisms-09-00574-s001.pdf]

Table S1. Assessment of the quality of the studies included in this review. Study quality was evaluated based on a scoring system attributing one point if the respective information was provided and zero points if it was missing. For studies with multiple study cohorts, a point was attributed if the information was available for at least one of these cohorts.

**Reference**	**Study year given**	**Study location given**	**Informed consent/ Ethic Approval**	**Exclusion criteria given**	**Sample size calculation done**	**Statistical software named**	**Test method/ manufacturer named**	**Cut off titer given**	**Specimen type provided**	**Age given**	**Overall result/ confidence interval given**	**Age-stratified seroprevalences provided**	**Bias named**	**Peer reviewed journal used**	**Total**
Richard-Lenoble et al., 1999 [1]	0	1	0/0	0	0	0	1/1	0	1	1	1/0	0	0	0	**6**
Priest et al., 2016 [2]	1	1	1/1	0	1	1	1/0	1	1	1	1/1	1	1	1	**15**
Catár et al., 1992 [3]	0	0	0/0	0	0	0	1/0	1	1	1	1/0	1	0	0	**6**
Andiappan et al., 2014 [4]	0	1	1/1	0	0	1	1/1	0	1	1	1/1	0	0	1	**11**
Hakim et al., 1994 [5]	0	1	0/0	0	0	1	1/0	1	1	0	1/0	1	0	0	**7**
Nissapatorn et al., 2002 [6]	0	1	1/0	0	0	1	1/0	0	1	1	1/0	1	1	1	**10**
Nissapatorn et al., 2003 [7]	1	1	0/0	0	0	1	1/1	1	0	1	1/0	0	0	1	**9**
Nissapatorn et al., 2004 [8]	1	1	0/0	0	0	1	1/1	1	0	1	1/1	0	0	1	**10**
Nissapatorn et al., 2003 [9]	1	1	1/0	0	0	1	1/1	1	1	1	1/1	1	0	0	**12**
Nissapatorn et al., 2003 [10]	1	1	1/0	1	0	1	1/1	1	1	1	1/1	1	0	1	**14**
Nissapatorn et al., 2005 [11]	1	1	0/0	0	0	1	1/1	0	0	1	1/0	1	0	0	**8**
Chan et al., 2008 [12]	0	1	0/0	0	0	0	1/0	1	1	0	1/0	0	0	0	**5**
Nissapatorn et al., 2011 [13]	1	1	1/1	1	0	1	1/1	1	1	1	1/1	1	0	0	**14**
Nimir et al., 2010 [14]	1	1	0/0	0	0	1	1/0	1	1	1	1/0	0	0	1	**9**
Ngui et al., 2011 [15]	1	0	1/1	0	0	1	1/1	1	1	1	1/1	1	0	1	**13**
Singh et al., 2012 [16]	1	1	1/1	1	0	1	1/1	1	1	1	1/1	1	1	1	**16**

Emelia et al., 2012 [17]	1	1	1/1	1	0	1	1/1	1	1	0	1/0	1	0	1	**13**
Omar et al., 2015 [18]	0	1	1/1	0	0	1	1/1	1	1	1	1/0	0	1	1	**12**
Ahmad et al., 2014 [19]	0	1	1/1	0	1	1	1/1	0	1	1	1/0	1	0	1	**12**
Emelia et al., 2014 [20]	1	1	1/1	1	0	1	1/1	1	1	0	1/0	0	0	1	**12**
Angal et al., 2016 [21]	1	1	1/1	0	0	1	1/1	1	1	0	1/0	0	0	1	**11**
Brandon-Mong et al., 2015 [22]	1	1	1/1	1	0	1	1/1	0	1	1	1/0	1	0	1	**13**
van Enter et al., 2017 [23]	1	1	0/1	0	0	1	1/1	1	1	1	1/1	0	0	1	**12**
Wong et al., 2000 [24]	1	1	0/0	0	0	1	1/0	1	1	0	1/0	0	0	1	**8**
Lim et al., 2013 [25]	1	1	0/1	0	0	1	0/0	1	0	0	1/0	0	1	1	**8**
Chintana et al., 1991 [26]	0	1	0/0	0	0	0	1/1	1	1	0	0/0	0	0	0	**5**
Taechowisan et al., 1997 [27]	1	1	0/0	0	0	0	1/1	0	1	1	0/0	0	0	1	**7**
Chintana et al., 1998 [28]	1	1	0/0	0	0	0	1/0	0	1	0	1/0	1	0	0	**6**
Sukthana, 1999 [29]	0	1	0/0	0	0	0	1/0	0	1	1	1/0	1	0	0	**6**
Maruyama et al., 2000 [30]	1	1	0/0	0	0	0	1/1	1	1	1	1/0	1	0	1	**10**
Pinlaor et al., 2000 [31]	1	1	0/0	0	0	1	1/1	1	1	1	1/0	1	0	0	**10**
Wanachiwanawin et al., 2001 [32]	1	1	1/0	0	1	0	1/1	0	1	0	1/0	0	0	1	**9**
Tantivanich et al., 2001 [33]	1	1	0/0	0	0	0	1/0	1	1	0	1/0	0	0	0	**6**
Sukthana et al., 2003 [34]	0	1	1/0	0	0	0	1/0	1	1	1	1/0	1	0	0	**8**
Sakae et al., 2013 [35]	1	1	1/1	0	0	0	1/1	1	1	1	1/0	1	0	1	**12**
Nissapatorn et al., 2011 [36]	1	1	1/1	1	0	1	1/1	1	1	1	1/1	1	0	1	**15**
Chemoh et al., 2013 [37]	1	1	1/1	0	0	1	1/0	1	1	0	1/0	0	0	1	**10**
Chemoh et al., 2015 [38]	1	1	1/1	0	0	1	1/1	0	1	1	1/0	1	0	1	**12**
Andiappan et al., 2014 [39]	1	1	1/1	0	0	1	1/1	0	1	1	1/1	1	0	1	**13**

Follézou et al., 1999 [40]	1	1	1/1	0	0	1	0/1	0	1	1	1/0	0	0	1	**10**
Udonsom et al., 2008 [41]	1	0	1/0	0	0	0	1/0	1	1	0	1/1	1	0	0	**8**
Buchy et al., 2003 [42]	0	0	0/0	0	0	0	1/1	0	1	1	1/0	0	0	0	**5**
Black et al., 2015 [43]	1	1	1/1	0	0	1	1/1	0	1	1	1/0	1	0	1	**12**
Yap et al., 2017 [44]dash	0	1	1/1	1	0	1	1/1	1	1	1	1/0	0	1	1	**13**
Dashraath et al., 2007 [45]	1	1	0/0	0	1	1	1/0	1	1	1	1/0	0	1	1	**11**
Fatha et al., 2014 [46]	1	1	1/1	1	1	1	1/1	1	1	1	1/1	0	1	1	**16**
Gorny et al., 2015 [47]	1	1	0/1	0	0	1	1/1	1	1	0	1/1	1	1	1	**13**
Migasena et al., 1997 [48]	1	1	1/0	0	0	0	1/1	1	1	1	1/0	1	0	1	**11**
Lolekha et al., 2001 [49]	1	1	1/1	1	0	1	1/1	1	1	1	1/1	1	0	1	**15**
Kowitdamrong et al., 2005 [50]	1	1	0/0	0	0	0	1/1	0	1	0	1/0	1	0	1	**8**
Srichomkwun et al., 2009 [51]	1	1	1/0	0	0	0	1/0	1	1	1	1/0	0	1	1	**10**
Suwanpakdee et al., 2012 [52]	1	1	1/0	0	0	1	1/1	0	1	1	1/0	0	0	1	**10**
Ooi et al., 2002 [53]	1	1	0/0	1	0	1	1/1	0	1	1	1/0	1	0	1	**11**
Matsunaga et al., 1994 [54]	1	0	0/0	0	0	0	1/0	0	1	0	1/0	0	1	1	**6**
Poovorawan et al., 2000 [55]	1	1	0/0	0	0	0	1/1	0	1	1	1/0	1	0	0	**8**
Suandork et al., 2000 [56]	1	1	1/0	1	0	0	1/1	0	1	1	1/0	1	0	1	**11**
Bhattarakosol et al., 2003 [57]	1	1	0/0	0	0	0	1/1	1	1	1	1/0	0	0	0	**8**
Mao et al., 2015 [58]	1	1	1/1	0	1	1	1/1	1	1	1	1/1	1	1	1	**16**
Phengxay et al., 2011 [59]	1	1	0/1	0	0	1	1/1	1	1	1	1/0	1	0	1	**12**
Hachiya et al., 2018 [60]	1	1	1/1	1	1	1	1/1	1	1	1	1/1	1	1	1	**17**
Sekawi et al., 2005 [61]	1	1	0/0	0	0	0	1/1	1	0	1	1/0	1	1	1	**10**
Cheong et al., 2008 [62]	1	1	1/1	1	1	1	1/1	1	1	1	1/0	0	1	1	**15**

Cheong et al., 2013 [63]	1	1	1/1	1	1	1	1/1	1	1	1	1/1	0	1	1	**16**
Ang et al., 2010 [64]	1	1	1/1	0	0	1	1/1	1	1	1	1/1	0	1	1	**14**
Liew et al., 2010 [65]	1	1	1/0	0	0	1	0/0	0	1	1	1/0	0	1	1	**9**
Chua et al., 2015 [66]	1	1	1/1	1	0	0	1/1	1	1	1	1/1	1	1	1	**15**
Ang et al., 2013 [67]	1	1	0/0	1	1	1	1/1	1	1	1	1/1	1	1	1	**15**
Boonruang et al., 2005 [68]	1	1	1/0	1	0	0	1/0	1	1	1	1/0	1	0	1	**11**
Tharmap-hornpilas et al., 2009 [69]	1	1	0/1	1	1	0	1/1	1	1	1	1/1	1	0	1	**14**
Chaiwarith et al., 2016 [70]	1	1	1/1	1	1	1	1/1	1	0	1	1/0	1	1	1	**15**
Sreepian et al., 2018 [71]	1	1	0/1	1	1	1	1/1	1	1	1	1/1	1	0	0	**14**
Miyakawa et al., 2014 [72]	1	1	1/1	1	0	1	1/1	1	1	1	1/1	1	1	1	**16**
Ahmed et al., 2006 [73]	0	1	1/0	0	0	0	1/1	1	1	1	1/0	0	0	0	**8**
Saraswathy et al., 2011 [74]	1	1	0/0	0	0	0	1/1	0	1	0	1/0	0	0	0	**6**
Urwijitaroon et al., 1993 [75]	1	0	0/0	0	0	0	1/1	0	1	1	1/0	1	0	0	**7**
Tantivanich et al., 1999 [76]	1	1	0/0	0	0	0	1/0	1	1	1	1/0	0	0	0	**7**
Amarapal et al., 2001 [77]	1	1	0/0	0	0	0	1/0	1	1	1	1/0	1	0	0	**8**
Fongsarun et al., 2013 [78]	0	0	0/0	0	0	1	0/1	1	1	0	1/0	0	0	1	**6**
Bhattarakoso l et al., 1998 [79]	1	1	0/0	0	0	1	1/1	0	1	1	0/0	1	0	0	**8**
O'Charoen et al., 1992 [80]	1	1	0/0	0	0	0	1/1	1	1	0	1/0	0	0	0	**7**
Theng et al., 2006 [81]	1	1	1/1	0	0	1	1/1	0	1	1	1/0	1	0	1	**12**
Theng et al., 2006 [82]	1	1	1/1	1	0	1	1/1	1	1	1	1/0	1	0	1	**14**
Nopkesorn et al., 1998 [83]	1	0	0/1	0	0	0	1/0	0	1	0	1/1	0	0	1	**7**
Dobbins et al., 1999 [84]	1	0	0/0	0	0	1	1/0	0	1	1	1/1	0	0	1	**8**

Limpakarnjanarat et al., 1999 [85]	1	1	1/1	1	0	1	1/0	0	1	0	1/0	0	0	1	**10**
Van Griensven et al., 2013 [86]	1	1	1/1	1	0	1	1/1	0	1	1	1/0	0	1	1	**13**
Ashley-Morrow et al., 2004 [87]	1	0	1/1	0	0	0	1/1	1	1	1	1/0	0	0	1	**10**
Bollen et al., 2008 [88]	1	1	1/1	0	0	0	1/1	0	1	1	1/0	0	1	1	**11**
Holtz et al., 2012 [89]	1	1	1/1	1	0	1	1/1	1	1	0	1/0	0	1	1	**13**
Le et al., 2009 [90]	1	1	1/1	1	0	0	1/1	1	1	1	1/1	1	1	1	**15**
Anh et al., 2003 [91]	1	0	1/1	1	0	0	1/1	0	1	0	1/0	0	0	1	**9**
Go et al., 2006 [92]	1	0	1/1	1	0	1	1/1	0	1	0	1/1	1	1	1	**13**
O'Farrell et al., 2006 [93]	1	0	1/1	0	0	1	0/1	0	0	0	1/1	0	0	1	**8**
Vu Thuong et al., 2007 [94]	1	1	1/1	0	1	1	0/0	0	0	0	1/0	0	0	1	**8**
Ngo et al., 2008 [95]	1	0	0/1	0	0	0	1/1	0	1	0	1/0	0	0	1	**7**
Nguyen et al., 2009 [96]	1	1	0/1	1	0	1	1/1	0	1	1	0/1	0	1	1	**12**
**Summary**	**81**	**82**	**54/50**	**30**	**14**	**59**	**90/71**	**59**	**88**	**69**	**92/30**	**49**	**28**	**71**	

References

1. Richard-Lenoble, D.; Cheng, H.K.; Sire, J.M.; Duong, T.H.; Cheng, T.V.; Phanny, I.; Rainsy, T.; Cauchoix, C. Toxoplasmosis in Cambodia : initial serological evaluation at Phnom Penh *Sante* **1999**, *9*, 377-382.
2. Priest, J.W.; Jenks, M.H.; Moss, D.M.; Mao, B.; Buth, S.; Wannemuehler, K.; Soeung, S.C.; Lucchi, N.W.; Udhayakumar, V.; Gregory, C.J., et al. Integration of Multiplex Bead Assays for Parasitic Diseases into a National, Population-Based Serosurvey of Women 15-39 Years of Age in Cambodia. *PLoS neglected tropical diseases* **2016**, *10*, e0004699, doi:10.1371/journal.pntd.0004699.
3. Catar, G.; Giboda, M.; Gutvirth, J.; Hongvanthong, B. Seroepidemiological study of toxoplasmosis in Laos. *The Southeast Asian journal of tropical medicine and public health* **1992**, *23*, 491-492.
4. Andiappan, H.; Nissapatorn, V.; Sawangjaroen, N.; Nyunt, M.H.; Lau, Y.L.; Khaing, S.L.; Aye, K.M.; Mon, N.C.; Tan, T.C.; Kumar, T., et al. Comparative study on Toxoplasma infection between Malaysian and Myanmar pregnant women. *Parasites & vectors* **2014**, *7*, 564, doi:10.1186/s13071-014-0564-9.
5. Hakim, S.L.; Radzan, T.; Nazma, M. Distribution of anti-Toxoplasma gondii antibodies among Orang Asli (aborigines) in Peninsular Malaysia. *The Southeast Asian journal of tropical medicine and public health* **1994**, *25*, 485-489.
6. Nissapatorn, V.; Kamarulzaman, A.; Init, I.; Tan, L.H.; Rohela, M.; Norliza, A.; Chan, L.L.; Latt, H.M.; Anuar, A.K.; Quek, K.F. Seroepidemiology of toxoplasmosis among HIV-infected patients and healthy blood donors. *The Medical journal of Malaysia* **2002**, *57*, 304-310.
7. Nissapatorn, V.; Lee, C.K.; Khairul, A.A. Seroprevalence of toxoplasmosis among AIDS patients in Hospital Kuala Lumpur, 2001. *Singapore medical journal* **2003**, *44*, 194-196.
8. Nissapatorn, V.; Lee, C.; Quek, K.F.; Leong, C.L.; Mahmud, R.; Abdullah, K.A. Toxoplasmosis in HIV/AIDS patients: a current situation. *Japanese journal of infectious diseases* **2004**, *57*, 160-165.
9. Nissapatorn, V.; Lee, C.K.; Cho, S.M.; Rohela, M.; Anuar, A.K.; Quek, K.F.; Latt, H.M. Toxoplasmosis in HIV/AIDS patients in Malaysia. *The Southeast Asian journal of tropical medicine and public health* **2003**, *34 Suppl 2*, 80-85.

10. Nissapatorn, V.; Noor Azmi, M.A.; Cho, S.M.; Fong, M.Y.; Init, I.; Rohela, M.; Khairul Anuar, A.; Quek, K.F.; Latt, H.M. Toxoplasmosis: prevalence and risk factors. *Journal of obstetrics and gynaecology : the journal of the Institute of Obstetrics and Gynaecology* **2003**, *23*, 618-624, doi:10.1080/01443610310001604376.
11. Nissapatorn, V.; Lim, Y.A.; Jamaiah, I.; Agnes, L.S.; Amyliana, K.; Wen, C.C.; Nurul, H.; Nizam, S.; Quake, C.T.; Valartmathi, C., et al. Parasitic infections in Malaysia: changing and challenges. *The Southeast Asian journal of tropical medicine and public health* **2005**, *36 Suppl 4*, 50-59.
12. Chan, B.T.; Amal, R.N.; Hayati, M.I.; Kino, H.; Anisah, N.; Norhayati, M.; Sulaiman, O.; Abdullah, M.M.; Fatmah, M.S.; Roslida, A.R., et al. Seroprevalence of toxoplasmosis among migrant workers from different Asian countries working in Malaysia. *The Southeast Asian journal of tropical medicine and public health* **2008**, *39*, 9-13.
13. Nissapatorn, V.; Leong, T.H.; Lee, R.; Init, I.; Ibrahim, J.; Yen, T.S. Seroepidemiology of toxoplasmosis in renal patients. *The Southeast Asian journal of tropical medicine and public health* **2011**, *42*, 237-247.
14. Nimir, A.; Othman, A.; Ee, S.; Musa, Z.; Majid, I.A.; Kamarudin, Z.; Xian, C.; Isa, N.H. Latent toxoplasmosis in patients with different malignancy: a hospital based study. *J Clin Med Res* **2010**, *2*, 117-120, doi:10.4021/jocmr2010.06.375w.
15. Ngui, R.; Lim, Y.A.; Amir, N.F.; Nissapatorn, V.; Mahmud, R. Seroprevalence and sources of toxoplasmosis among Orang Asli (indigenous) communities in Peninsular Malaysia. *The American journal of tropical medicine and hygiene* **2011**, *85*, 660-666, doi:10.4269/ajtmh.2011.11-0058.
16. Singh, S.; Khang, T.F.; Andiappan, H.; Nissapatorn, V.; Subrayan, V. An age-adjusted seroprevalence study of Toxoplasma antibody in a Malaysian ophthalmology unit. *Transactions of the Royal Society of Tropical Medicine and Hygiene* **2012**, *106*, 322-326, doi:10.1016/j.trstmh.2012.01.009.
17. Emelia, O.; Amal, R.N.; Ruzanna, Z.Z.; Shahida, H.; Azzubair, Z.; Tan, K.S.; Noor Aadila, S.; Siti, N.A.; Aisah, M.Y. Seroprevalence of anti-Toxoplasma gondii IgG antibody in patients with schizophrenia. *Tropical biomedicine* **2012**, *29*, 151-159.
18. Omar, A.; Bakar, O.C.; Adam, N.F.; Osman, H.; Osman, A.; Suleiman, A.H.; Manaf, M.R.; Selamat, M.I. Seropositivity and serointensity of Toxoplasma gondii antibodies and DNA among patients with schizophrenia. *The Korean journal of parasitology* **2015**, *53*, 29-34, doi:10.3347/kjp.2015.53.1.29.
19. Ahmad, A.F.; Ngui, R.; Muhammad Aidil, R.; Lim, Y.A.; Rohela, M. Current status of parasitic infections among Pangkor Island community in Peninsular Malaysia. *Tropical biomedicine* **2014**, *31*, 836-843.
20. Emelia, O.; Rahana, A.R.; Mohamad Firdaus, A.; Cheng, H.S.; Nursyairah, M.S.; Fatinah, A.S.; Azmawati, M.N.; Siti, N.A.; Aisah, M.Y. IgG avidity assay: a tool for excluding acute toxoplasmosis in prolonged IgM titer sera from pregnant women. *Tropical biomedicine* **2014**, *31*, 633-640.
21. Angal, L.; Lim, Y.A.; Yap, N.J.; Ngui, R.; Amir, A.; Kamarulzaman, A.; Rohela, M. Toxoplasmosis in HIV and non HIV prisoners in Malaysia. *Tropical biomedicine* **2016**, *33*, 159–169.
22. Brandon-Mong, G.J.; Che Mat Seri, N.A.; Sharma, R.S.; Andiappan, H.; Tan, T.C.; Lim, Y.A.; Nissapatorn, V. Seroepidemiology of Toxoplasmosis among People Having Close Contact with Animals. *Frontiers in immunology* **2015**, *6*, 143, doi:10.3389/fimmu.2015.00143.
23. van Enter, B.J.D.; Lau, Y.L.; Ling, C.L.; Watthanaworawit, W.; Sukthana, Y.; Lee, W.C.; Nosten, F.; McGready, R. Seroprevalence of Toxoplasma gondii Infection in Refugee and Migrant Pregnant Women along the Thailand-Myanmar Border. *The American journal of tropical medicine and hygiene* **2017**, *97*, 232-235, doi:10.4269/ajtmh.16-0999.
24. Wong, A.; Tan, K.H.; Tee, C.S.; Yeo, G.S. Seroprevalence of cytomegalovirus, toxoplasma and parvovirus in pregnancy. *Singapore medical journal* **2000**, *41*, 151-155.
25. Lim, R.B.; Tan, M.T.; Young, B.; Lee, C.C.; Leo, Y.S.; Chua, A.; Ng, O.T. Risk factors and time-trends of cytomegalovirus (CMV), syphilis, toxoplasmosis and viral hepatitis infection and seroprevalence in human immunodeficiency virus (HIV) infected patients. *Annals of the Academy of Medicine, Singapore* **2013**, *42*, 667-673.
26. Chintana, T. Pattern of antibodies in toxoplasmosis of pregnant women and their children in Thailand. *The Southeast Asian journal of tropical medicine and public health* **1991**, *22 Suppl*, 107-110.
27. Taechowisan, T.; Sutthent, R.; Louisirirotchanakul, S.; Puthavathana, P.; Wasi, C. Immune status in congenital infections by TORCH agents in pregnant Thais. *Asian Pacific journal of allergy and immunology* **1997**, *15*, 93-97.
28. Chintana, T.; Sukthana, Y.; Bunyakai, B.; Lekkla, A. Toxoplasma gondii antibody in pregnant women with and without HIV infection. *The Southeast Asian journal of tropical medicine and public health* **1998**, *29*, 383-386.
29. Sukthana, Y. Difference of Toxoplasma gondii antibodies between Thai and Austrian pregnant women. *The Southeast Asian journal of tropical medicine and public health* **1999**, *30*, 38-41.
30. Maruyama, S.; Boonmar, S.; Morita, Y.; Sakai, T.; Tanaka, S.; Yamaguchi, F.; Kabeya, H.; Katsube, Y. Seroprevalence of Bartonella henselae and Toxoplasma gondii among healthy individuals in Thailand. *The Journal of veterinary medical science* **2000**, *62*, 635-637.
31. Pinlaor, S.; Ieamviteevanich, K.; Pinlaor, P.; Maleewong, W.; Pipitgool, V. Seroprevalence of specific total immunoglobulin (Ig), IgG and IgM antibodies to Toxoplasma gondii in blood donors from Loei Province, Northeast Thailand. *The Southeast Asian journal of tropical medicine and public health* **2000**, *31*, 123-127.

32. Wanachiwanawin, D.; Sutthent, R.; Chokephaibulkit, K.; Mahakittikun, V.; Ongrotchanakun, J.; Monkong, N. Toxoplasma gondii antibodies in HIV and non-HIV infected Thai pregnant women. *Asian Pacific journal of allergy and immunology* **2001**, *19*, 291-293.
33. Tantivanich, S.; Amarapal, P.; Suphadtanaphongs, W.; Siripanth, C.; Sawatmongkonkun, W. Prevalence of congenital cytomegalovirus and Toxoplasma antibodies in Thailand. *The Southeast Asian journal of tropical medicine and public health* **2001**, *32*, 466-469.
34. Sukthana, Y.; Kaewkungwal, J.; Jantanavivat, C.; Lekkla, A.; Chiabchalard, R.; Aumarm, W. Toxoplasma gondii antibody in Thai cats and their owners. *The Southeast Asian journal of tropical medicine and public health* **2003**, *34*, 733-738.
35. Sakae, C.; Natphopsuk, S.; Settheetham-Ishida, W.; Ishida, T. Low prevalence of Toxoplasma gondii infection among women in northeastern Thailand. *The Journal of parasitology* **2013**, *99*, 172-173, doi:10.1645/GE-3222.1.
36. Nissapatorn, V.; Suwanrath, C.; Sawangjaroen, N.; Ling, L.Y.; Chandeying, V. Toxoplasmosis-serological evidence and associated risk factors among pregnant women in southern Thailand. *The American journal of tropical medicine and hygiene* **2011**, *85*, 243-247, doi:10.4269/ajtmh.2011.10-0633.
37. Chemoh, W.; Sawangjaroen, N.; Nissapatorn, V.; Suwanrath, C.; Chandeying, V.; Hortiwakul, T.; Andiappan, H.; Sermwittayawong, N.; Charoenmak, B.; Siripaitoon, P., et al. Toxoplasma gondii infection: What is the real situation? *Experimental parasitology* **2013**, *135*, 685-689, doi:10.1016/j.exppara.2013.10.001.
38. Chemoh, W.; Sawangjaroen, N.; Siripaitoon, P.; Andiappan, H.; Hortiwakul, T.; Sermwittayawong, N.; Charoenmak, B.; Nissapatorn, V. Toxoplasma gondii - Prevalence and Risk Factors in HIV-infected Patients from Songklanagarind Hospital, Southern Thailand. *Frontiers in microbiology* **2015**, *6*, 1304, doi:10.3389/fmicb.2015.01304.
39. Andiappan, H.; Nissapatorn, V.; Sawangjaroen, N.; Chemoh, W.; Lau, Y.L.; Kumar, T.; Onichandran, S.; Suwanrath, C.; Chandeying, V. Toxoplasma infection in pregnant women: a current status in Songklanagarind hospital, southern Thailand. *Parasites & vectors* **2014**, *7*, 239, doi:10.1186/1756-3305-7-239.
40. Follezou, J.Y.; Lan, N.Y.; Lien, T.X.; Lafon, M.E.; Tram, L.T.; Hung, P.V.; Aknine, X.; Lowenstein, W.; Ngai, N.V.; Theodorou, I., et al. Clinical and biological characteristics of human immunodeficiency virus-infected and uninfected intravascular drug users in Ho Chi Minh City, Vietnam. *The American journal of tropical medicine and hygiene* **1999**, *61*, 420-424.
41. Udonsom, R.; Lekkla, A.; Chung, P.T.; Cam, P.D.; Sukthana, Y. Seroprevalence of Toxoplasma gondii antibody in Vietnamese villagers. *The Southeast Asian journal of tropical medicine and public health* **2008**, *39*, 14-18.
42. Buchy, P.; Follezou, J.Y.; Lien, T.X.; An, T.T.; Tram, L.T.; Tri, D.V.; Cuong, N.M.; Glaziou, P.; Chien, B.T. [Serological study of toxoplasmosis in Vietnam in a population of drug users (Ho Chi Minh city) and pregnant women (Nha Trang)]. *Bulletin de la Societe de pathologie exotique* **2003**, *96*, 46-47.
43. Black, A.P.; Vilivong, K.; Nouanthong, P.; Souvannaso, C.; Hubschen, J.M.; Muller, C.P. Serosurveillance of vaccine preventable diseases and hepatitis C in healthcare workers from Lao PDR. *PloS one* **2015**, *10*, e0123647, doi:10.1371/journal.pone.0123647.
44. Yap, S.H.; Abdullah, N.K.; McStea, M.; Takayama, K.; Chong, M.L.; Crisci, E.; Larsson, M.; Azwa, I.; Kamarulzaman, A.; Leong, K.H., et al. HIV/Human herpesvirus co-infections: Impact on tryptophan-kynurenine pathway and immune reconstitution. *PloS one* **2017**, *12*, e0186000, doi:10.1371/journal.pone.0186000.
45. Dashraath, P.; Ong, E.S.; Lee, V.J. Seroepidemiology of varicella and the reliability of a self-reported history of varicella infection in Singapore military recruits. *Annals of the Academy of Medicine, Singapore* **2007**, *36*, 636-641.
46. Fatha, N.; Ang, L.W.; Goh, K.T. Changing seroprevalence of varicella zoster virus infection in a tropical city state, Singapore. *International journal of infectious diseases : IJID : official publication of the International Society for Infectious Diseases* **2014**, *22*, 73-77, doi:10.1016/j.ijid.2013.10.003.
47. Gorny, A.W.; Mittal, C.; Saw, S.; Venkatachalam, I.; Fisher, D.A.; Tambyah, P.A. Varicella seroprevalence in healthcare workers in a tertiary hospital: an audit of cross-sectional data. *BMC research notes* **2015**, *8*, 664, doi:10.1186/s13104-015-1656-0.
48. Migasena, S.; Simasathien, S.; Desakorn, V.; Phonrat, B.; Suntharasamai, P.; Pitisuttitham, P.; Aree, C.; Naksrisook, S.; Supeeranun, L.; Samakoses, R., et al. Seroprevalence of Varicella-Zoster Virus Antibody in Thailand. *International journal of infectious diseases : IJID : official publication of the International Society for Infectious Diseases* **1997**, *2*, 26-30.
49. Lolekha, S.; Tanthiphabha, W.; Sornchai, P.; Kosuwan, P.; Sutra, S.; Warachit, B.; Chup-Upprakarn, S.; Hutagalung, Y.; Weil, J.; Bock, H.L. Effect of climatic factors and population density on varicella zoster virus epidemiology within a tropical country. *The American journal of tropical medicine and hygiene* **2001**, *64*, 131-136.
50. Kowitdamrong, E.; Pancharoen, C.; Thammaborvorn, R.; Bhattarakosol, P. The prevalence of varicella-zoster virus infection in normal healthy individuals aged above 6 months. *Journal of the Medical Association of Thailand = Chotmaihet thangphaet* **2005**, *88 Suppl 4*, S7-11.

51. Srichomkwun, P.; Apisarnthanarak, A.; Thongphubeth, K.; Yuekyen, C.; Mundy, L.M. Evidence of vaccine protection among thai medical students and implications for occupational health. *Infection control and hospital epidemiology* **2009**, *30*, 585-588, doi:10.1086/597508.
52. Suwanpakdee, D.; Laohapand, C.; Moolasart, V.; Lomtong, P.; Krairojananan, N.; Srisawat, P.; Watanaveeradej, V. Serosurveillance of varicella and hepatitis B infection after reported cases in medical students and the relationship between past varicella disease history and immunity status. *Journal of the Medical Association of Thailand = Chotmaihet thangphaet* **2012**, *95 Suppl 5*, S80-85.
53. Ooi, S.L.; Hooi, P.S.; Chua, B.H.; Lam, S.K.; Chua, K.B. Seroprevalence of human parvovirus B19 infection in an urban population in Malaysia. *The Medical journal of Malaysia* **2002**, *57*, 97-103.
54. Matsunaga, Y.; Goh, K.T.; Utagawa, E.; Muroi, N. Low prevalence of antibody to human parvovirus B19 in Singapore. *Epidemiology and infection* **1994**, *113*, 537-540.
55. Poovorawan, Y.; Theamboonlers, A.; Suandork, P.; Hirsch, P. Prevalence of antibodies to parvovirus B 19 in Thailand. *The Southeast Asian journal of tropical medicine and public health* **2000**, *31*, 422-424.
56. Suandork, P.; Theamboonlers, A.; Likitnukul, S.; Hirsch, P.; Poovorawan, Y. Parvovirus B19 antibodies in immunocompromized children in Thailand. *Asian Pacific journal of allergy and immunology* **2000**, *18*, 161-164.
57. Bhattarakosol, P.; Pancharoen, C.; Kowitdamrong, E.; Thammaborvorn, R.; Mungmee, V. Prevalence of parvovirus B19 infection in Thai young adults. *The Southeast Asian journal of tropical medicine and public health* **2003**, *34*, 585-588.
58. Mao, B.; Chheng, K.; Wannemuehler, K.; Vynnycky, E.; Buth, S.; Soeung, S.C.; Reef, S.; Weldon, W.; Quick, L.; Gregory, C.J. Immunity to polio, measles and rubella in women of child-bearing age and estimated congenital rubella syndrome incidence, Cambodia, 2012. *Epidemiology and infection* **2015**, *143*, 1858-1867, doi:10.1017/S0950268814002817.
59. Phengxay, M.; Hayakawa, Y.; Phan, T.G.; Uneno-Yamamoto, K.; Tanaka-Taya, K.; Vongphrachanh, P.; Komase, K.; Ushijima, H. Seroprevalence of rubella and measles antibodies in Lao PDR. *Clinical laboratory* **2011**, *57*, 237-244.
60. Hachiya, M.; Miyano, S.; Mori, Y.; Vynnycky, E.; Keungsaneth, P.; Vongphrachanh, P.; Xeuatvongsa, A.; Sisouk, T.; Som-Oulay, V.; Khamphaphongphane, B., et al. Evaluation of nationwide supplementary immunization in Lao People's Democratic Republic: Population-based seroprevalence survey of anti-measles and anti-rubella IgG in children and adults, mathematical modelling and a stability testing of the vaccine. *PloS one* **2018**, *13*, e0194931, doi:10.1371/journal.pone.0194931.
61. Sekawi, Z.; Muizatul, W.M.; Marlyn, M.; Jamil, M.A.; Ilina, I. Rubella vaccination programme in Malaysia: analysis of a seroprevalence study in an antenatal clinic. *The Medical journal of Malaysia* **2005**, *60*, 345-348.
62. Cheong, A.T.; Khoo, E.M. Prevalence of rubella susceptibility among pregnant mothers in a community-based antenatal clinic in Malaysia: a cross-sectional study. *Asia-Pacific journal of public health* **2008**, *20*, 340-346, doi:10.1177/1010539508322698.
63. Cheong, A.T.; Tong, S.F.; Khoo, E.M. How useful is a history of rubella vaccination for determination of disease susceptibility? A cross-sectional study at a public funded health clinic in Malaysia. *BMC family practice* **2013**, *14*, 19, doi:10.1186/1471-2296-14-19.
64. Ang, L.W.; Chua, L.T.; James, L.; Goh, K.T. Epidemiological surveillance and control of rubella in Singapore, 1991-2007. *Annals of the Academy of Medicine, Singapore* **2010**, *39*, 95-101.
65. Liew, F.; Ang, L.W.; Cutter, J.; James, L.; Goh, K.T. Evaluation on the effectiveness of the national childhood immunisation programme in Singapore, 1982-2007. *Annals of the Academy of Medicine, Singapore* **2010**, *39*, 532-510.
66. Chua, Y.X.; Ang, L.W.; Low, C.; James, L.; Cutter, J.L.; Goh, K.T. An epidemiological assessment towards elimination of rubella and congenital rubella syndrome in Singapore. *Vaccine* **2015**, *33*, 3150-3157, doi:10.1016/j.vaccine.2015.04.003.
67. Ang, L.W.; Lai, F.Y.; Tey, S.H.; Cutter, J.; James, L.; Goh, K.T. Prevalence of antibodies against measles, mumps and rubella in the childhood population in Singapore, 2008-2010. *Epidemiology and infection* **2013**, *141*, 1721-1730, doi:10.1017/S0950268812002130.
68. Boonruang, S.; Buppasiri, P. Rubella antibodies in normal pregnant women at Srinagarind Hospital, Khon Kaen, Thailand. *Journal of the Medical Association of Thailand = Chotmaihet thangphaet* **2005**, *88*, 455-459.
69. Tharmaphornpilas, P.; Yoocharean, P.; Rasdjarmrearnsook, A.O.; Theamboonlers, A.; Poovorawan, Y. Seroprevalence of antibodies to measles, mumps, and rubella among Thai population: evaluation of measles/MMR immunization programme. *Journal of health, population, and nutrition* **2009**, *27*, 80-86.
70. Chaiwarith, R.; Praparattanapan, J.; Nuket, K.; Kotarathitithum, W.; Supparatpinyo, K. Seroprevalence of antibodies to measles, mumps, and rubella, and serologic responses after vaccination among human immunodeficiency virus (HIV)-1 infected adults in Northern Thailand. *BMC infectious diseases* **2016**, *16*, 190, doi:10.1186/s12879-016-1499-x.
71. Sreepian, P.M.; Sreepian, A. Seroprevalence of Rubella Immunity among women of childbearing age in Bangkok, Thailand. *The Southeast Asian journal of tropical medicine and public health* **2018**, *49*, 76-81.

72. Miyakawa, M.; Yoshino, H.; Yoshida, L.M.; Vynnycky, E.; Motomura, H.; Tho le, H.; Thiem, V.D.; Ariyoshi, K.; Anh, D.D.; Moriuchi, H. Seroprevalence of rubella in the cord blood of pregnant women and congenital rubella incidence in Nha Trang, Vietnam. *Vaccine* **2014**, *32*, 1192-1198, doi:10.1016/j.vaccine.2013.08.076.
73. Ahmed, S.A.; Al-Joudi, F.S.; Zaidah, A.W.; Roshan, T.M.; Rapiaah, M.; Abdullah, Y.M.; Rosline, H. The prevalence of human cytomegalovirus seropositivity among blood donors at the Unit of Blood Transfusion Medicine, Hospital Universiti Sains Malaysia. *The Southeast Asian journal of tropical medicine and public health* **2006**, *37*, 294-296.
74. Saraswathy, T.S.; Az-Ulhusna, A.; Asshikin, R.N.; Suriani, S.; Zainah, S. Seroprevalence of cytomegalovirus infection in pregnant women and associated role in obstetric complications: a preliminary study. *The Southeast Asian journal of tropical medicine and public health* **2011**, *42*, 320-322.
75. Urwijitaroon, Y.; Teawpatanataworn, S.; Kitjareontarm, A. Prevalence of cytomegalovirus antibody in Thai-northeastern blood donors. *The Southeast Asian journal of tropical medicine and public health* **1993**, *24 Suppl 1*, 180-182
76. Tantivanich, S.; Suphadtanaphongs, V.; Siripanth, C.; Desakorn, V.; Suphanit, I.; Phromin, S.; Panakitsuwan, S.; Amarapand, P. Prevalence of cytomegalovirus antibodies among various age groups of Thai population. *The Southeast Asian journal of tropical medicine and public health* **1999**, *30*, 265-268.
77. Amarapal, P.; Tantivanich, S.; Balachandra, K. Prevalence of cytomegalovirus in Thai blood donors by monoclonal staining of blood leukocytes. *The Southeast Asian journal of tropical medicine and public health* **2001**, *32*, 148-153.
78. Fongsarun, J.E., Maneerat; Paisan, Mantana; Chanthachorn, Siripen and Papadopoulos, Konstantinos I. Prevalence of transmissible viral disease in maternal blood samples of autologous umbilical cord blood in a private cord blood bank. *Transplantation Technology* **2013**, 10.7243/2053-6623-1-1, doi:10.7243/2053-6623-1-1.
79. Bhattarakosol, P.; Sithidajporn, M.; Bhattarakosol, P. Seroprevalence of cytomegalovirus infection in Thai adults detecting by ELISA. *Chula Med J* **1998**.
80. O'Charoen; Nuchprayoon, C.; Chumnijarakij, T.; Ganapi, S. Cytomegalovirus antibody screening program of Thai blood donors for bone-marrow transplant patients. **1992** Theng, T.S.; Sen, P.R.; Tan, H.H.; Wong, M.L.; Chan, K.W. Seroprevalence of HSV-1 and 2 among sex workers attending a sexually transmitted infection clinic in Singapore. *International journal of STD & AIDS* **2006**, *17*, 395-399, doi:10.1258/095646206777323364.
81. Theng, T.S.; Sen, P.R.; Tan, H.H.; Wong, M.L.; Chan, K.W. Seroprevalence of HSV-1 and 2 among sex workers attending a sexually transmitted infection clinic in Singapore. *International journal of STD & AIDS* **2006**, *17*, 395-399, doi:10.1258/095646206777323364.
82. Theng, C.T.; Sen, P.R.; Chio, T.W.; Tan, H.H.; Wong, M.L.; Chan, R.K. Seroprevalence of herpes simplex virus-1 and -2 in attendees of a sexually transmitted infection clinic in Singapore. *Sexual health* **2006**, *3*, 269-274.
83. Nopkesorn, T.; Mock, P.A.; Mastro, T.D.; Sangkharomya, S.; Sweat, M.; Limpakarnjanarat, K.; Laosakkitiboran, J.; Young, N.L.; Morse, S.A.; Schmid, S., et al. HIV-1 subtype E incidence and sexually transmitted diseases in a cohort of military conscripts in northern Thailand. *Journal of acquired immune deficiency syndromes and human retrovirology : official publication of the International Retrovirology Association* **1998**, *18*, 372-379.
84. Dobbins, J.G.; Mastro, T.D.; Nopkesorn, T.; Sangkharomya, S.; Limpakarnjanarat, K.; Weniger, B.G.; Schmid, D.S. Herpes in the time of AIDS: a comparison of the epidemiology of HIV-1 and HSV-2 in young men in northern Thailand. *Sexually transmitted diseases* **1999**, *26*, 67-74.
85. Limpakarnjanarat, K.; Mastro, T.D.; Saisorn, S.; Uthaivoravit, W.; Kaewkungwal, J.; Korattana, S.; Young, N.L.; Morse, S.A.; Schmid, D.S.; Weniger, B.G., et al. HIV-1 and other sexually transmitted infections in a cohort of female sex workers in Chiang Rai, Thailand. *Sexually transmitted infections* **1999**, *75*, 30-35.
86. van Griensven, F.; Thienkrua, W.; McNicholl, J.; Wimonsate, W.; Chaikummao, S.; Chonwattana, W.; Varangrat, A.; Sirivongrangson, P.; Mock, P.A.; Akarasewi, P., et al. Evidence of an explosive epidemic of HIV infection in a cohort of men who have sex with men in Thailand. *Aids* **2013**, *27*, 825-832, doi:10.1097/QAD.0b013e32835c546e.
87. Ashley-Morrow, R.; Nollkamper, J.; Robinson, N.J.; Bishop, N.; Smith, J. Performance of focus ELISA tests for herpes simplex virus type 1 (HSV-1) and HSV-2 antibodies among women in ten diverse geographical locations. *Clinical microbiology and infection : the official publication of the European Society of Clinical Microbiology and Infectious Diseases* **2004**, *10*, 530-536, doi:10.1111/j.1469-0691.2004.00836.x.
88. Bollen, L.J.; Whitehead, S.J.; Mock, P.A.; Leelawiwat, W.; Asavapiriyanont, S.; Chalermchockchareonkit, A.; Vanprapar, N.; Chotpitayasunondh, T.; McNicholl, J.M.; Tappero, J.W., et al. Maternal herpes simplex virus type 2 coinfection increases the risk of perinatal HIV transmission: possibility to further decrease transmission? *Aids* **2008**, *22*, 1169-1176, doi:10.1097/QAD.0b013e3282fec42a.
89. Holtz, T.H.; Thienkrua, W.; McNicholl, J.M.; Wimonsate, W.; Chaikummao, S.; Chonwattana, W.; Wasinrapee, P.; Varangrat, A.; Mock, P.A.; Sirivongrangson, P., et al. Prevalence of Treponema pallidum seropositivity and herpes simplex virus type 2 infection in a cohort of men who have sex with men, Bangkok, Thailand, 2006-2010. *International journal of STD & AIDS* **2012**, *23*, 424-428, doi:10.1258/ijsa.2011.011256.

90. Le, H.V.; Schoenbach, V.J.; Herrero, R.; Hoang Pham, A.T.; Nguyen, H.T.; Nguyen, T.T.; Munoz, N.; Franceschi, S.; Vaccarella, S.; Parkin, M.D., et al. Herpes simplex virus type-2 seropositivity among ever married women in South and north Vietnam: a population-based study. *Sexually transmitted diseases* **2009**, *36*, 616-620, doi:10.1097/OLQ.0b013e3181a8cde4.
91. Anh, P.T.H.; Hieu, N.T.; Herrero, R.; Vaccarella, S.; Smith, J.S.; Nguyen Thuy, T.T.; Nguyen, H.N.; Nguyen, B.D.; Ashley, R.; Snijders, P.J., et al. Human papillomavirus infection among women in South and North Vietnam. *Int J Cancer* **2003**, *104*, 213-220, doi:10.1002/ijc.10936.
92. Go, V.F.; Frangakis, C.; Nam le, V.; Bergenstrom, A.; Sripaipan, T.; Zenilman, J.M.; Celentano, D.D.; Quan, V.M. High HIV sexual risk behaviors and sexually transmitted disease prevalence among injection drug users in Northern Vietnam: implications for a generalized HIV epidemic. *Journal of acquired immune deficiency syndromes* **2006**, *42*, 108-115, doi:10.1097/01.qai.0000199354.88607.2f.
93. O'Farrell, N.; Thuong, N.V.; Nghia, K.V.; Tram, L.T.; Long, N.T. HSV-2 antibodies in female sex workers in Vietnam. *International journal of STD & AIDS* **2006**, *17*, 755-758, doi:10.1258/095646206778691176.
94. Vu Thuong, N.; Van Nghia, K.; Phuc Hau, T.; Thanh Long, N.; Thi Bao Van, C.; Hoang Duc, B.; Thu Tram, L.; Anh Tuan, N.; Thi Kim Tien, N.; Godwin, P., et al. Impact of a community sexually transmitted infection/HIV intervention project on female sex workers in five border provinces of Vietnam. *Sexually transmitted infections* **2007**, *83*, 376-382, doi:10.1136/sti.2006.022616.
95. Ngo, T.D.; Laeyendecker, O.; La, H.; Hogrefe, W.; Morrow, R.A.; Quinn, T.C. Use of commercial enzyme immunoassays to detect antibodies to the herpes simplex virus type 2 glycoprotein G in a low-risk population in Hanoi, Vietnam. *Clinical and vaccine immunology : CVI* **2008**, *15*, 382-384, doi:10.1128/CVI.00437-06.
96. Nguyen, N.T.; Nguyen, H.T.; Trinh, H.Q.; Mills, S.J.; Detels, R. Clients of female sex workers as a bridging population in Vietnam. *AIDS and behavior* **2009**, *13*, 881-891, doi:10.1007/s10461-008-9463-4.
